# Systematic evaluation of tools for auxin-inducible protein degradation in budding yeast

**DOI:** 10.1091/mbc.E25-12-0571

**Published:** 2025-12-16

**Authors:** Petra Hubbe, Charu Sharma, Oliver Pajonk, Niklas Peters, Nadja Guschtschin-Schmidt, Natalie Friemel, Sebastian Schuck

**Affiliations:** ^1^Heidelberg University Biochemistry Center, 69120 Heidelberg, Germany

## Abstract

The auxin system for inducible protein degradation is a powerful tool to investigate protein function. It consists of a degron fused to a target protein, an auxin-related ligand that binds to the degron, and a receptor that recognizes the auxin-bound degron and mediates proteasomal degradation of the target protein. Variants of all system components are available, and we here test three degrons, three auxins and three degron receptors to identify optimal combinations of these variants in budding yeast. We show that the degrons mIAA7 or AID* together with adamantyl-auxin and the degron receptor OsTIR1(F74G) allow particularly rapid and extensive degradation. Basal degradation in the absence of auxin is generally low and can be minimized further by inducible expression of OsTIR1(F74G). Moreover, we demonstrate that the remarkable efficiency of this system makes it competitive with established chemical inhibitors, such as tunicamycin and MG132, and with temperature-sensitive conditional alleles. These findings will aid the effective application of the auxin system.

## Abstract (executive summary)

- The auxin system for inducible protein degradation is a powerful tool for investigating protein function in budding yeast. Variants of all system components are available, but which ones should be combined for optimal results is unclear.
- The authors systematically evaluated various system components and found that the degrons mIAA7 or AID* together with adamantyl-auxin and the degron receptor OsTIR1(F74G) allow particularly rapid and extensive protein depletion, with little basal degradation. Moreover, they demonstrated that the system can replace chemical inhibitors and temperature-sensitive alleles.
- This study clarifies how the auxin system should best be used and will aid its effective application.

## INTRODUCTION

Biological research often aims to define the physiological functions of individual proteins. The most direct approach for this purpose is to inactivate a protein of interest and examine the resulting cellular or organismal phenotypes. Ideally, protein inactivation should be specific, complete and immediate. Non-essential proteins can be removed through knockout of the corresponding genes, which is specific and complete, but slow. It takes many cell division cycles until newly created mutant cells can be analyzed so that cell adaptation may gradually obscure the knockout phenotype. Similar limitations apply when the mRNA of a target protein is eliminated, for instance through RNA interference, because the impact on protein function depends on how quickly pre-existing protein molecules are degraded. If a target protein is long-lived, its levels will decline only slowly. In the meantime, cell adaptation may again alter the observed phenotype. Given these caveats, target protein inactivation should be as fast as possible.

Several systems are available for rapid target protein inactivation in eukaryotic cells by inducible degradation (Prozzillo *et al.*, 2020). In budding yeast, *Saccharomyces cerevisiae*, target protein degradation can be induced by drug addition, temperature shift, or light (Kanemaki *et al.*, 2003; Nishimura *et al.*, 2009; Hasenjäger *et al.*, 2019). The only option currently available for drug-inducible protein degradation is the auxin system. This system is based on the plant hormone indole-3-acetic acid, which binds to Aux/IAA proteins and mediates their interaction with F-box proteins of the TIR1/AFB family. In this way, indole-3-acetic acid triggers recognition of Aux/IAA proteins by TIR1/AFB-containing ubiquitin ligase complexes and subsequent degradation of Aux/IAA by the proteasome (Leyser, 2018). The system can be transferred to non-plant cells. To this end, a TIR1/AFB protein is introduced into the cells of interest and an Aux/IAA protein is fused to a target protein by genome editing. Aux/IAA then serves as a conditional degradation determinant (called degron) so that the target protein is degraded upon addition of indole-3-acetic acid (Nishimura *et al.*, 2009). This transfer across species is possible because the plant TIR1/AFB proteins can be incorporated into native SCF ubiquitin ligase complexes in many different organisms (Phanindhar and Mishra, 2023).

All components of the auxin system have been improved through engineering efforts, yielding different Aux/IAA variants (degrons), indole-3-acetic acid derivatives (auxins) and TIR1/AFB variants (degron receptors). These improvements have reduced degron size, restricted basal degradation in the absence of auxin, lowered the auxin concentration and thus avoided off-target effects, and increased the speed of target protein degradation. A key advance was the implementation of a bump-and-hole strategy, in which the addition of a bulky chemical moiety to auxin and a corresponding enlargement of the auxin binding pocket of degron receptors through amino acid exchanges strengthened the interaction between auxin-bound degron and receptor (Uchida *et al.*, 2018; Yamada *et al.*, 2018; Nishimura *et al.*, 2020). Various system components are now available, raising the question of which ones should be combined for optimal target protein degradation.

**FIGURE 1:**
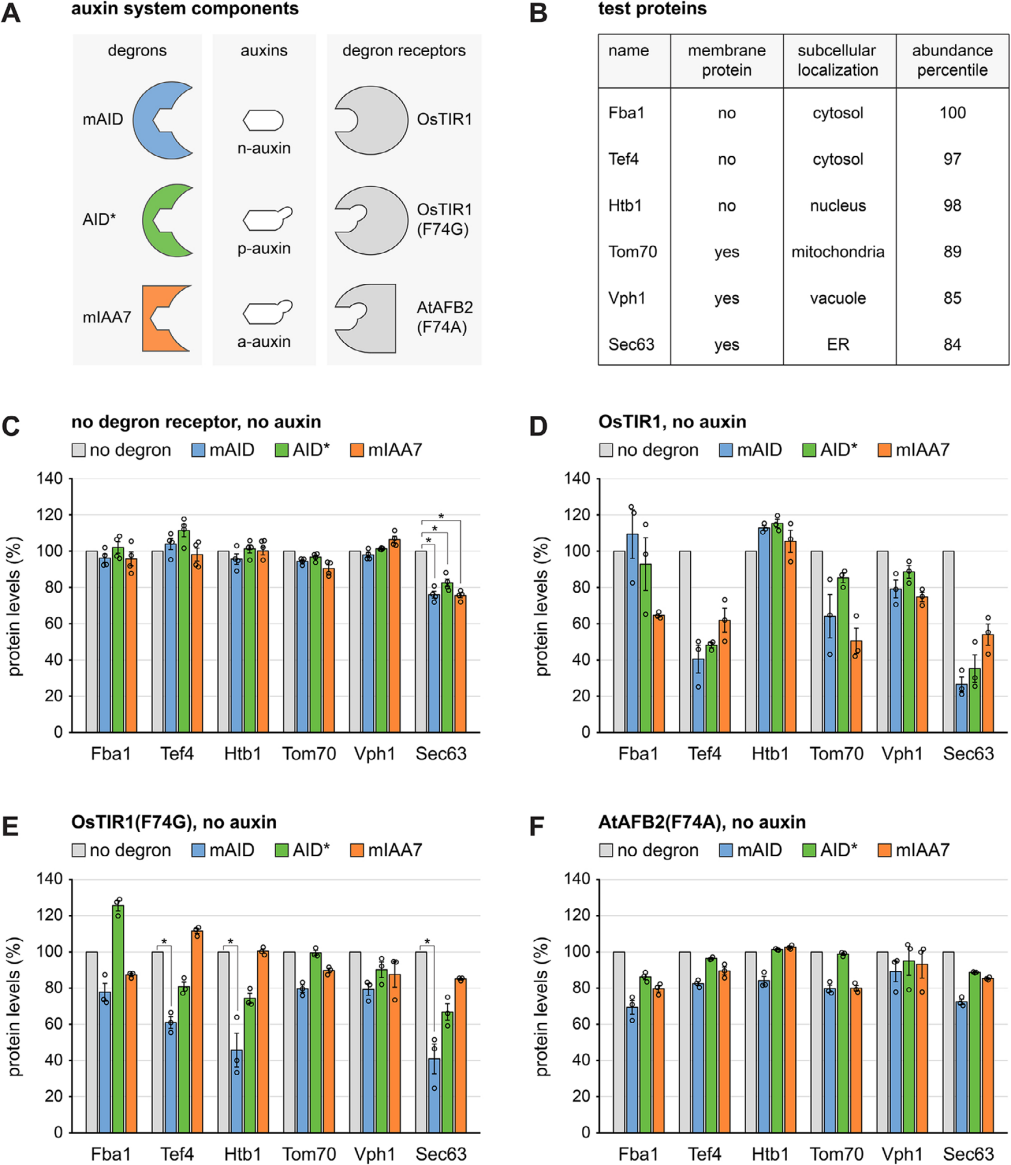
Auxin system components, test proteins and basal degradation in the absence of auxin. (A) Degrons, auxins and degron receptors used in this study. mAID (8 kDa) and AID* (5 kDa) are derived from AtIAA17; mIAA7 (8 kDa) is derived from AtIAA7. N-auxin is the naturally occurring indole-3-acetic acid, p-auxin, and a-auxin contain additional phenyl and adamantyl moieties, respectively. OsTIR1 is a naturally occurring degron receptor, OsTIR1(F74G) and AtAFB2(F74A) are mutant variants in which the auxin binding pocket is enlarged to accommodate the artificial auxins p-auxin and a-auxin. (B) Test proteins used to evaluate combinations of degron, auxin and degron receptor. Cellular levels of these proteins are in the 84th–100th abundance percentile of all yeast proteins detectable by mass spectrometry (Platzek *et al.*, 2025). (C) Flow cytometry measurement of relative levels of test proteins tagged with mNeonGreen (no degron), mAID-mNeonGreen (mAID), AID*-mNeonGreen (AID*), or mIAA7-mNeonGreen (mIAA7). Cells did not express a degron receptor and were not exposed to auxin. Bars show the mean protein levels of *n* = 4 biological replicates. Error bars show the standard error of the mean. Protein levels were normalized to the levels in cells expressing the respective test protein tagged only with mNeonGreen. * *p*<0.05 as calculated with a two-tailed heteroscedastic *t* test. (D-F) As in panel C but in cells expressing OsTIR1, OsTIR1(F74G), and AtAFB2(F74A), respectively. Data are from *n* = 3 biological replicates.

Here, we systematically evaluated combinations of three degrons, three auxins, and three degron receptors in budding yeast (Figure 1A). The degrons were the shortened AtIAA17 variants mini-auxin inducible degron (mAID, 8 kDa in size; Kubota *et al.*, 2013) and AID* (5 kDa in size; Morawska and Ulrich, 2013), and the shortened AtIAA7 variant mini-IAA7 (mIAA7; 8 kDa in size; Li *et al.*, 2019). The inducers were the natural indole-3-acetic acid and its artificial derivatives 5-phenyl- and 5-adamantyl-indole-3-acetic acid (Uchida *et al.*, 2018; Yamada *et al.*, 2018). These auxins have been abbreviated IAA, 5-Ph-IAA, and 5-Ad-IAA, but for simplicity we here refer to them as natural auxin, phenyl-auxin and adamantyl-auxin, or n-auxin, p-auxin and a-auxin for short. The degron receptors were the natural OsTIR1 and the OsTIR1(F74G) and AtAFB(F74A) variants with enlarged auxin binding pockets (Nishimura *et al.*, 2009; Yesbolatova *et al.*, 2020; Li *et al.*, 2024). We show that the combination of mIAA7, a-auxin, and OsTIR1(F74G) allows extensive and rapid inducible protein degradation, with minimal basal degradation. Furthermore, we demonstrate that this system can be competitive with established chemical inhibitors and temperature-sensitive conditional alleles.

## RESULTS

### Basal protein degradation by degron tagging and expression of degron receptors

To assess combinations of degrons, auxins and degron receptors, we determined basal and auxin-induced degradation of six test proteins. This set consisted of soluble and transmembrane proteins from different subcellular compartments. Specifically, we chose (1) the cytosolic glycolytic enzyme Fba1, (2) the cytosolic translation elongation factor Tef4, (3) the nuclear histone Htb1, (4) the mitochondrial outer membrane protein Tom70, (5) the vacuolar membrane protein Vph1, and (6) the endoplasmic reticulum (ER) membrane protein Sec63 (Figure 1B). The localizations and topologies of these proteins ensure that degrons fused to their C-termini are exposed to the cytosol or nucleoplasm and are thus accessible to the ubiquitin-proteasome system. Furthermore, all six proteins are among the 20% most abundant proteins in yeast (Platzek *et al.*, 2025). These high levels were chosen to challenge the capacity of the auxin-inducible degradation system.

We first asked whether fusion of the degrons to the test proteins caused degradation in the absence of auxins or degron receptors. We used chromosomal gene tagging to generate strains in which the C-termini of test proteins were fused to the fluorescent protein mNeonGreen or to cassettes consisting of mNeonGreen and the degrons mAID, AID* or mIAA7. Quantification of cellular mNeonGreen fluorescence by flow cytometry showed that the test proteins were not generally destabilized by the addition of the degrons. An exception was Sec63, whose levels were lowered by about 20% upon fusion to degron-mNeonGreen cassettes compared with fusion to mNeonGreen alone (Figure 1C). Furthermore, fusion of mAID-mNeonGreen to Htb1 yielded yeast with a severe growth defect, indicating that Htb1-mAID-mNeonGreen was functionally impaired (Supplemental Figure S1A). We then tested whether auxins changed the abundance of degron-tagged test proteins in the absence of a degron receptor, but they did not (Supplemental Figure S1, C–H). Hence, tagging a target protein with one of the degrons is unlikely to cause severe destabilization in the absence of a degron receptor, even upon auxin treatment. However, there may be exceptions and the tagged protein may not be fully functional.

Next, we determined whether expression of any of the three degron receptors led to basal degradation of degron-tagged proteins in the absence of auxins. Expression of OsTIR1 under the constitutive *ADH* promoter had only minor destabilizing effects on degron-tagged Fba1, Htb1 and Vph1. However, OsTIR1 reduced the levels of degron-tagged Tef4, Tom70, and Sec63 to varying extents, which could exceed 50% (Figure 1D). These results are consistent with previously reported basal degradation of degron-tagged proteins by OsTIR1, which likely arises from auxin-independent association of OsTIR1 with auxin-binding degrons (Yesbolatova *et al.*, 2020). In addition, many yeast strains produce n-auxin (Liu *et al.*, 2016), which may contribute to basal degradation. Expression of OsTIR1(F74G) or AtAFB2(F74A) had overall smaller destabilizing effects on degron-tagged test proteins (Figure 1, D–F; Supplemental Figure S1B). Hence, these receptor variants alleviate the problem of basal degradation, as reported (Yesbolatova *et al.*, 2020; Li *et al.*, 2024). We therefore excluded OsTIR1 from further analysis. However, the mAID degron together with OsTIR1(F74G) still caused substantial basal degradation of Tef4, Htb1, and Sec63 (Figure 1E). By contrast, the AID* and mIAA7 degrons in combination with OsTIR1(F74G) and AtAFB2(F74A) generally caused only minor basal degradation (Figure 1, E–F).

### Auxin-inducible protein degradation by different combinations of system components

**FIGURE 2:**
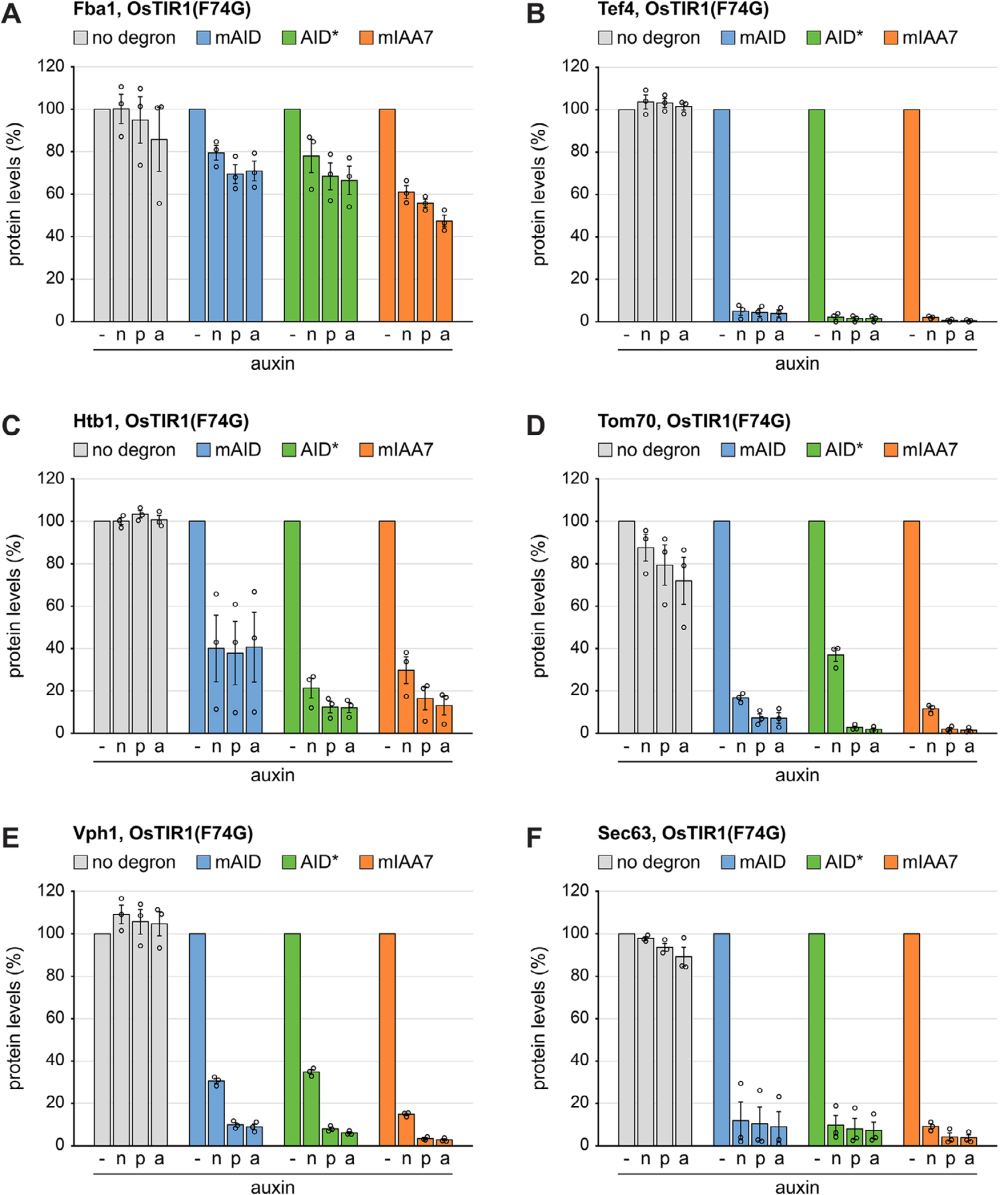
Inducible protein degradation by n-/p-/a-auxin in the presence of OsTIR1(F74G). (A) Flow cytometry measurement of relative levels of Fba1 tagged with mNeonGreen (no degron), mAID-mNeonGreen (mAID), AID*-mNeonGreen (AID*), or mIAA7-mNeonGreen (mIAA7). Cells expressed OsTIR1(F74G) and were either not treated or treated with 375 µM n-auxin, 5 µM p-auxin or 0.5 µM a-auxin for 120 min. Bars show the mean protein levels of *n* = 3 biological replicates, error bars show the standard error of the mean. Protein levels were normalized to the levels in untreated cells. (B-F) As in panel A but for Tef4, Htb1, Tom70, Vph1, and Sec63, respectively.

We then turned to auxin-induced protein degradation and compared the different auxins in end-point measurements. Guided by previous studies, we used n-auxin at 375 µM, p-auxin at 5 µM, and a-auxin at 0.5 µM (Yesbolatova *et al.*, 2020; Yamada *et al.*, 2018). The extent of inducible degradation after two hours differed between test proteins and also depended on the degron and the degron receptor (Figure 2; Supplemental Figure S2). For example, Fba1, which belongs to the 10 most abundant proteins in yeast (Platzek *et al.*, 2025), showed only modest degradation. By contrast, Tef4 and Sec63 levels were reduced by at least 80%, regardless of the degron and degron receptor used. However, across all proteins, p-auxin and a-auxin yielded more extensive degradation than n-auxin (differences between n-auxin and the other two auxin derivatives significant at the 5% level as determined from the data in Figure 2 and Supplemental Figure S2 with a one-way ANOVA followed by a Tukey test). In addition, a-auxin performed at least as well as p-auxin, even though its concentration was 10-fold lower. A direct comparison of a-auxin and p-auxin used at the same concentrations confirmed that a-auxin was superior (Supplemental Figure S3, A and B). This result is consistent with a-auxin having a higher affinity for OsTIR1(F74G) than p-auxin (Yamada *et al.*, 2018). Furthermore, we titrated the a-auxin concentration across a 50-fold range from 0.1 to 5 µM. Concentrations above 0.5 µM slightly improved degradation of the highly abundant Htb1 but had little effect on the less abundant Vph1 (Supplemental Figure S3, C and D). We used a-auxin at 0.5 µM in subsequent experiments but we note that, depending on the target protein, raising the a-auxin concentration can enhance degradation.

**FIGURE 3:**
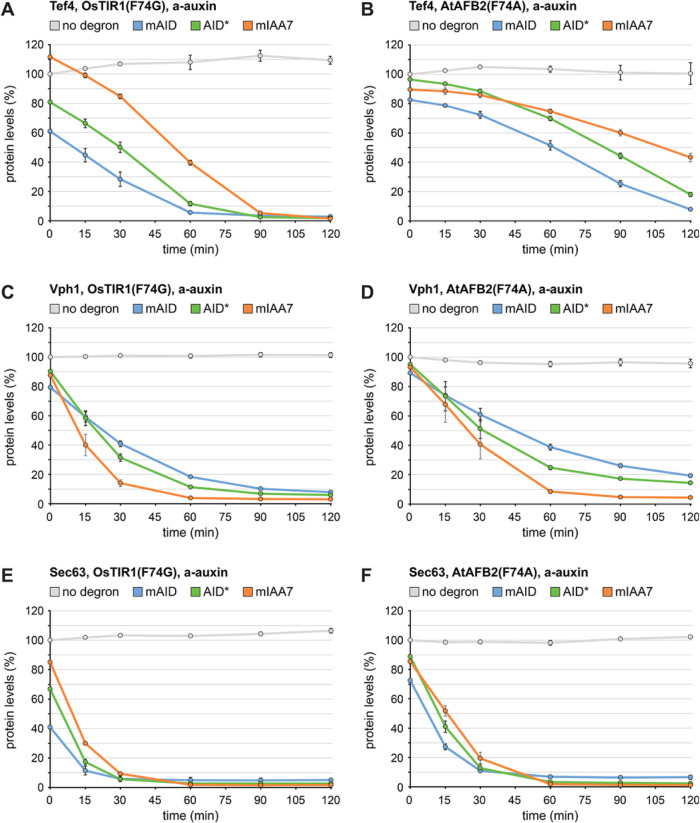
Kinetics of inducible protein degradation by a-auxin in the presence of OsTIR1(F74G) or AtAFB2(F74A). (A) Flow cytometry measurement of relative levels of Tef4 tagged with mNeonGreen (no degron), mAID-mNeonGreen (mAID), AID*-mNeonGreen (AID*) or mIAA7-mNeonGreen (mIAA7). Cells expressed OsTIR1(F74G) and were treated with 0.5 µM a-auxin for 0, 15, 30, 60, 90, or 120 min. Lines show the mean protein levels of *n* = 3 biological replicates, error bars show the standard error of the mean. Protein levels were normalized to the levels in cells that expressed Tef4-mNeonGreen and were treated with a-auxin for 0 min. (B) As in panel A but for cells expressing AtAFB2(F74A). (C, E) As in panel A but for cells expressing tagged variants of Vph1 and Sec63, respectively. (D, F) As in panel A but for cells expressing AtAFB2(F74A) and tagged variants of Vph1, and Sec63, respectively.

As a next step, we asked which degron and degron receptor worked best. The end-point measurements above suggested that OsTIR1(F74G) was superior to AtAFB2(F74A). The point mutations OsTIR1(F74A) and AtAFB2(F74G) have also been tried (Yesbolatova *et al.*, 2020; Li *et al.*, 2024) and we confirmed that they offered no advantage over OsTIR1(F74G) and AtAFB2(F74A), respectively (Supplemental Figure S3E). In addition, we tested the recently described OsTIR1(F74G,S210A) variant (Xing *et al.*, 2025). However, it also did not perform better than OsTIR1(F74G) with regards to basal degradation or degradation efficiency (Supplemental Figure S3, F and G). We then carried out time-course measurements to follow the degradation of degron-tagged test proteins by a-auxin in the presence of OsTIR1(F74G) or AtAFB2(F74A). For each protein, we normalized its fluorescence in strains with various degrons to its fluorescence in strains in which it was tagged only with mNeonGreen. The resulting plots visualize basal degradation as differences between protein levels at the 0-min time point and show auxin-induced degradation as a decline in protein levels up to the 120-min time point (Figure 3; Supplemental Figure S4, A–F). As expected, degradation of Fba1 was inefficient. Depletion was at most 50%, which was achieved by OsTIR1(F74G) together with mAID or mIAA7 (Supplemental Figure S4, A and B). The other proteins were degraded more efficiently, and degradation rates correlated inversely with protein abundance (Supplemental Figure S4, G and H). Specifically, the order of degradation rates was Htb1 < Tef4 << Vph1 < Tom70 ≈ Sec63, while the order of abundance was Htb1 > Tef4 >> Tom70 > Vph1 ≈ Sec63. Furthermore, OsTIR1(F74G) yielded faster degradation than AtAFB2(F74A) across proteins and degrons (*p* = 0.0008, calculated from the data plotted in Supplemental Figure S4G, H with a two-tailed paired *t* test; also refer to Figure 3 and Supplemental Figure S4, A–F). The performance of the three degrons in the presence of OsTIR1(F74G) was similar but mIAA7 showed the least basal degradation (refer to Tef4, Sec63 and Htb1 in Figure 3, A and E and Supplemental Figure S4C). Overall, basal levels of degron-tagged proteins in the presence of OsTIR1(F74G) deviated from the levels of proteins tagged only with mNeonGreen by 36 ± 19% for mAID, 19 ± 12% for AID* and 10 ± 6% for mIAA7 (mean ± SD; differences between mAID and the other two degrons significant at the 1% level as determined with a one-way ANOVA followed by a Tukey test). Thus, mIAA7 together with OsTIR1(F74G) provided the best combination of low basal degradation and rapid auxin-inducible degradation. Remarkably, a-auxin treatment of cells expressing OsTIR1(F74G) reduced the levels of mIAA7-tagged Tom70, Vph1 and Sec63 by approximately 90% within 30 min and by >95% within 60 min (Supplemental Figure S4E; Figure 3, C and E). Even Tef4, which is among the 100 most abundant proteins in yeast (out of about 5400 proteins present; Ho *et al.*, 2018), was depleted by >95% within 120 min (Figure 3A).

In conclusion, the three degrons together with a-auxin and OsTIR1(F74G) enable rapid and extensive inducible degradation, also of very abundant proteins. Considering the generally low basal degradation seen with mIAA7 and OsTIR1(F74G), we chose the mIAA7/a-auxin/OsTIR1(F74G) system for further investigation.

### Impact of OsTIR1(F74G) levels on target protein degradation

The results above indicated that the extent of auxin-induced degradation declined with increasing target protein abundance. A possible reason for this trend is that the levels of the degron receptor limit the capacity of the auxin system. In this case, raising OsTIR1(F74G) levels should enhance the degradation of abundant target proteins. On the other hand, strong OsTIR1(F74G) expression may disturb the ubiquitin-proteasome system because OsTIR1(F74G) competes with other F-box proteins for incorporation into ubiquitin ligase complexes and could thereby interfere with the regular turnover of endogenous proteins. In this case, low OsTIR1(F74G) levels would be desirable. We therefore tested the effect of OsTIR1(F74G) abundance on target protein degradation.

**FIGURE 4:**
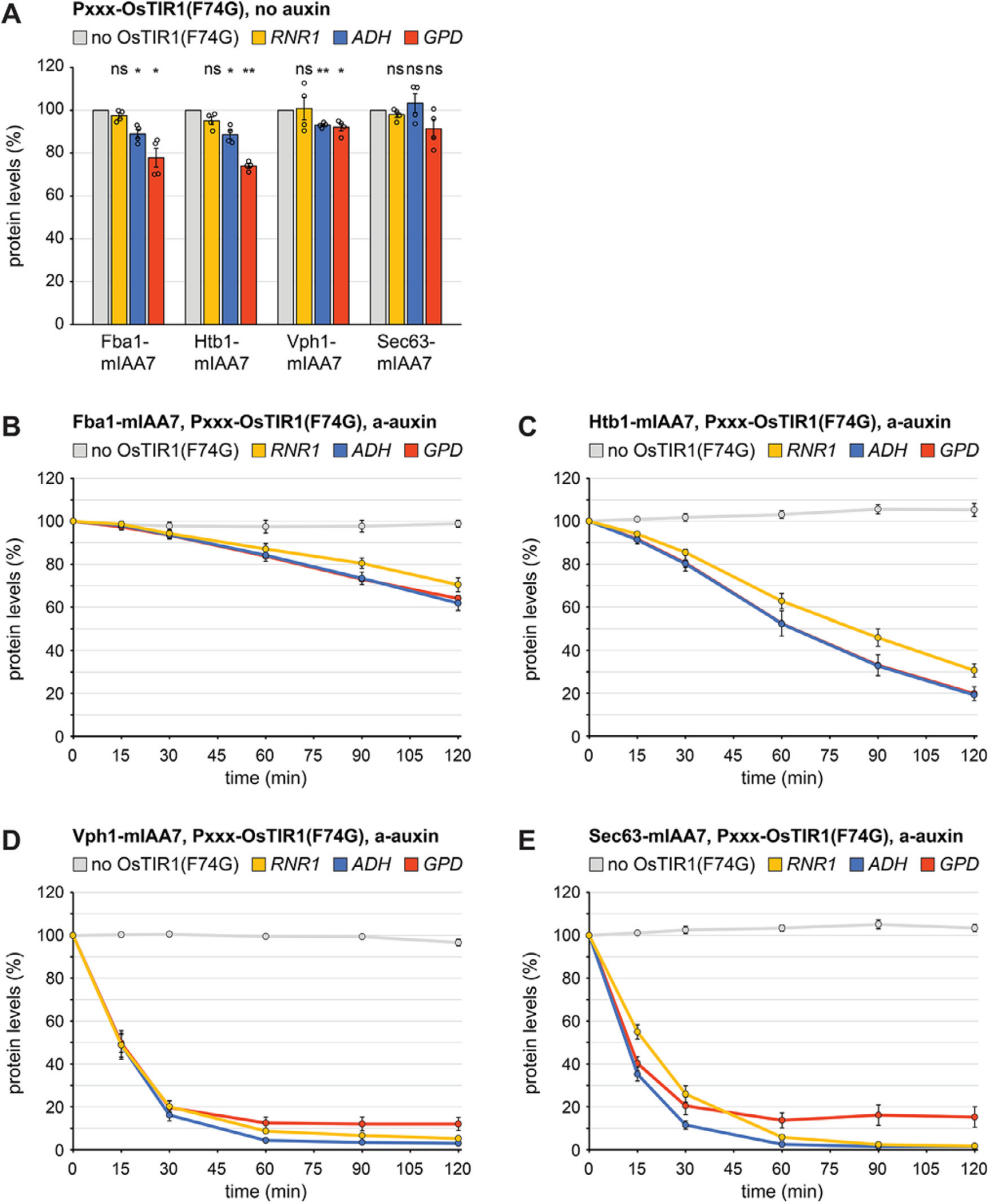
Basal and auxin-induced protein degradation by OsTIR1(F74G) expressed via different constitutive promoters. (A) Flow cytometry measurement of the levels of Fba1, Htb1, Vph1, and Sec63 tagged with mIAA7-mNeonGreen (mIAA7) in cells expressing no OsTIR1(F74G) or expressing OsTIR1(F74G) under the control of different constitutive promoters (P_XXX_), i.e. the weak *RNR1* promoter, the moderate *ADH* promoter or the very strong *GPD* promoter. Bars show the mean protein levels of *n* = 4 biological replicates, error bars show the standard error of the mean. Protein levels were normalized to the levels in cells not expressing OsTIR1(F74G). Statistical significance of the differences to the respective protein levels in cells without OsTIR1(F74G) was evaluated with a two-tailed heteroscedastic *t* test. * *p*<0.05, ** *p*<0.01, ns = not significant. (B) Flow cytometry measurement of Fba1-mIAA7-mNeonGreen (Fba1-mIAA7) levels in cells expressing no OsTIR1(F74G) or expressing OsTIR1(F74G) under the control of different constitutive promoters. Cells were treated with 0.5 µM a-auxin for 0, 15, 30, 60, 90, or 120 min. Lines show the mean protein levels of *n* = 4 biological replicates, error bars show the standard error of the mean. For each strain, protein levels were normalized to the levels at *t* = 0 min. (C–E) As in panel B but for Htb1, Vph1, and Sec63, respectively.

We placed OsTIR1(F74G) under the control of three different promoters, the weak *RNR1* promoter, the moderate *ADH* promoter used in all previous experiments, and the extremely strong *GPD* promoter (Supplemental Figure S5A). The resulting OsTIR1(F74G) protein levels matched the relative strength of the promoters (Supplemental Figure S5B). Basal degradation of mIAA7-tagged target proteins increased with increasing OsTIR1(F74G) abundance, at least for Fba1, Htb1 and Vph1 (Figure 4A). Auxin-induced degradation of Fba1, Htb1, Vph1, and Sec63 was faster when OsTIR1(F74G) expression was controlled by the *ADH* rather than the *RNR1* promoter (Figure 4, D–E). These results agree with a recent study that compared protein degradation by OsTIR1(F74G) expressed under the control of different constitutive promoters (Fülleborn *et al.*, 2025). However, the differences between the *ADH* and the *RNR1* promoter were minor, and *RNR1*-driven expression was sufficient for almost complete elimination of Sec63. The exceptionally strong *GPD* promoter did not outperform the *ADH* promoter, and in fact gave less complete degradation of Vph1 and Sec63. Hence, excessive OsTIR1(F74G) levels should be avoided, and OsTIR1(F74G) levels do not limit target protein degradation when the *ADH* promoter is used. The *RNR1* promoter is also a viable choice unless highly abundant proteins are targeted.

An alternative way to regulate OsTIR1(F74G) abundance is to use an inducible promoter. This format allows OsTIR1(F74G) to be expressed only at the time of auxin treatment. If the promoter is also titratable, OsTIR1(F74G) levels can be adjusted to the abundance of a target protein. We placed OsTIR1(F74G) under the control of the *GAL* promoter and the artificial transcription factor Gal4-ER-Msn2 (GEM), which activates the *GAL* promoter when the exogenous sterol β-estradiol is added. Furthermore, expression can be adjusted by varying the estradiol concentration (Pincus *et al.*, 2014; Schmidt *et al.*, 2019). To evaluate this system, we first used it to express superfolder GFP (sfGFP). Baseline sfGFP levels in the absence of estradiol were nearly undetectable, and overnight induction with saturating estradiol concentrations yielded levels similar to those achieved by the *ADH* promoter (Supplemental Figure S5A). Kinetic measurements showed that sfGFP levels reached those provided by the *RNR1* promoter within 1 h of induction and those provided by the *ADH* promoter within 3 h (Supplemental Figure S5C). Analysis of OsTIR1(F74G) by Western blotting confirmed these trends (Supplemental Figure S5B). Hence, the *GEM-GAL* system shows low baseline expression and allows robust gene induction.

**FIGURE 5:**
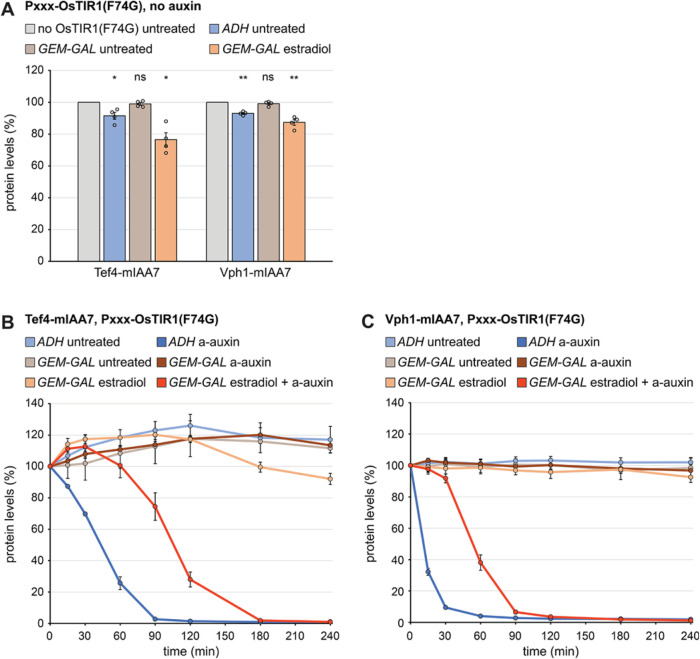
Basal and auxin-induced protein degradation by OsTIR1(F74G) expressed with the estradiol-inducible *GEM-GAL* promoter system. (A) Flow cytometry measurement of the levels of Tef4 and Vph1 tagged with mIAA7-mNeonGreen (mIAA7) in cells expressing no OsTIR1(F74G) or expressing OsTIR1(F74G) under the control of the constitutive *ADH* promoter or the inducible *GEM-GAL* promoter system. Cells were left untreated or treated with 800 nM estradiol for 16 h. Bars show the mean protein levels of *n* = 4 biological replicates, error bars show the standard error of the mean. Protein levels were normalized to the levels in cells not expressing OsTIR1(F74G). Statistical significance of the differences to the respective protein levels in cells without OsTIR1(F74G) was evaluated with a two-tailed heteroscedastic *t* test. * *p*<0.05, ** *p*<0.01, ns = not significant. (B) Flow cytometry measurement of Tef4-mIAA7-mNeonGreen (Tef4-mIAA7) levels in cells expressing OsTIR1(F74G) under the control of the *ADH* promoter or the *GEM-GAL* system. Cells were left untreated or treated with 800 nM estradiol and/or 0.5 µM a-auxin for 0, 15, 30, 60, 90, or 120 min. Lines show the mean protein levels of *n* = 3 biological replicates, error bars show the standard error of the mean. For each strain, protein levels were normalized to the levels at *t* = 0 min. (C) As in panel B but for Vph1.

Long-term OsTIR1(F74G) expression with the *GEM-GAL* system resulted in basal degradation even stronger to than seen with the *ADH* promoter. However, essentially no basal degradation was seen when the system was not induced (Figure 5A). We then combined estradiol-induced expression of OsTIR1(F74G) with the *GEM-GAL* system and auxin-induced protein degradation. Cells in which mIAA7-mNeonGreen was fused to Tef4 or Vph1 and which expressed OsTIR1(F74G) under the control of the *ADH* promoter or the *GEM-GAL* system were treated with auxin, with estradiol, or with both at the same time (Figure 5, B and C). In cells constitutively expressing OsTIR1(F74G), auxin reduced Tef4 and Vph1 levels by more than 90% within 80 and 30 min, respectively, as observed before (refer to Figure 3, A and C). In cells expressing OsTIR1(F74G) by means of the *GEM-GAL* system, there was no degradation upon treatment with auxin or estradiol alone, but the combined treatment reduced Tef4 and Vph1 levels by more than 90% within 160 and 90 min, respectively. Thus, adding the step of estradiol-induced synthesis of OsTIR1(F74G) delays target protein degradation by roughly 1 h. If this delay needs to be avoided, cells can first be treated with estradiol to induce OsTIR1(F74G) expression, and auxin can then be added to trigger target protein degradation.

In summary, OsTIR1(F74G) expression levels can be adjusted, both constitutively and inducibly, to tune them to target protein abundance. This approach minimizes the risk of perturbing the ubiquitin-proteasome system or causing basal target protein degradation.

### Application of the mIAA7/a-auxin/OsTIR1(F74G) system for inhibition of cellular activities

Next, we asked whether rapid protein degradation with the mIAA7/a-auxin/OsTIR1(F74G) system is comparable to protein inactivation with chemical inhibitors or temperature-sensitive (*ts*) alleles. We show that the effects of two widely used chemical inhibitors and a well-characterized *ts* allele can be recapitulated by auxin-inducible depletion of proteins acting in the same cellular pathways.

**FIGURE 6:**
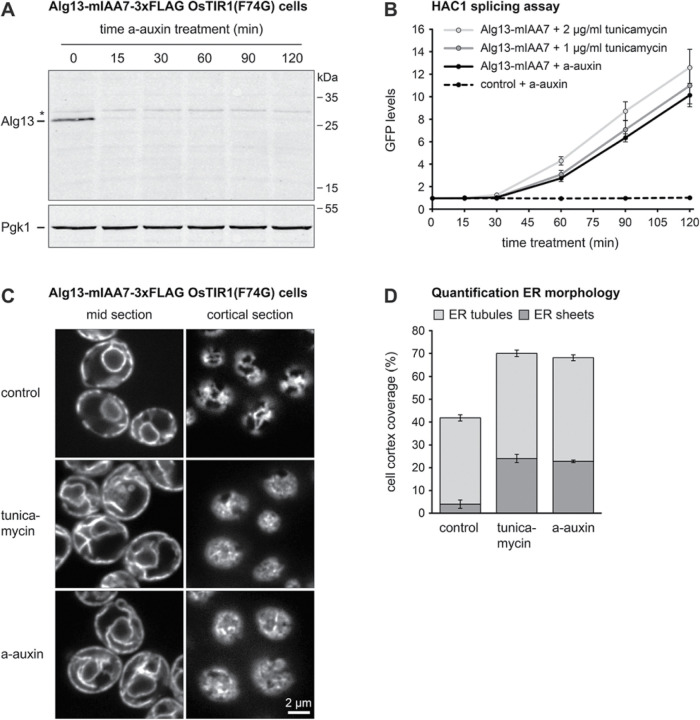
Induction of the unfolded protein response by tunicamycin and auxin-induced Alg13 depletion. (A) Western blot of FLAG tag from Alg13-mIAA7-3xFLAG OsTIR1(F74G) cells after treatment with 0.5 µM a-auxin for the times indicated. Pgk1 served as a loading control. The asterisk marks a non-specific band. The experiment was done once. (B) Flow cytometry measurement of GFP produced by the *HAC1* splicing reporter in OsTIR1(F74G) cells (control) and Alg13-mIAA7-3xFLAG OsTIR1(F74G) cells (Alg13-mIAA7) after treatment with 1 µg/ml tunicamycin, 2 µg/ml tunicamycin or 0.5 µM a-auxin. Lines show the mean GFP levels of *n* = 3 biological replicates, error bars show the standard error of the mean. (C) Sec63-mNeon images of confocal mid and cortical sections of Sec63-mNeon Alg13-mIAA7-3xFLAG OsTIR1(F74G) cells that were untreated (control), treated with 1 µg/ml tunicamycin or treated with 0.5 µM a-auxin. Tunicamycin-induced ER stress and auxin-induced Alg13 depletion cause ER expansion. The experiment was done twice. (D) Quantification of peripheral ER structures from images as in panel C. Bars are the mean percentage of cell cortex covered by tubules or sheets from 12 fields of view per condition, each containing at least 40 cells. Error bars show the 95% confidence interval.

Tunicamycin inhibits N-linked protein glycosylation and is commonly used to induce ER stress. Protein N-glycosylation requires the synthesis of a core oligosaccharide, which is initially anchored in the cytoplasmic leaflet of the ER membrane, is then translocated into the ER lumen, and is finally transferred onto nascent glycoproteins. The first step of core oligosaccharide synthesis is catalyzed by Alg7, the molecular target of tunicamycin (Barnes *et al.*, 1984). Alg7 is a multipass ER transmembrane protein, but cannot be detected at the ER by microscopy when a GFP tag is fused to its C-terminus (Dubreuil et al, 2019). Hence, it is unclear whether Alg7 tolerates C-terminal tagging. We therefore targeted the UDP-GlcNAc transferase Alg13 instead, which catalyzes the second step of core oligosaccharide synthesis and is a cytosolic protein peripherally associated with the ER membrane (Gao *et al.*, 2005). We fused mIAA7-3xFLAG to the C-terminus of Alg13 to enable auxin-inducible degradation. Western blotting showed that auxin reduced Alg13 abundance to almost undetectable levels within 15 min (Figure 6A). Alg13 is a low-abundance protein (Platzek *et al.*, 2025), which likely explains its very rapid depletion.

To assay the consequences of Alg13 degradation, we used a *HAC1* splicing reporter. When N-glycosylation is inhibited, newly synthesized glycoproteins fail to acquire their glycans and cannot fold properly. The resulting accumulation of misfolded proteins in the ER lumen causes ER stress and activates the unfolded protein response (UPR; Walter and Ron, 2011). This signaling pathway is controlled by the ER-resident endoribonuclease Ire1, which, upon ER stress, initiates splicing of the cytosolic *HAC1* mRNA. The *HAC1* splicing reporter is based on the *HAC1* mRNA but translates Ire1 activation into the production of GFP, which can be measured by flow cytometry (Pincus *et al.*, 2010). Tagging of Alg13 with mIAA7-3xFLAG by itself did not cause *HAC1* splicing (Figure 6B, control versus Alg13-mIAA7 strains at the 0-min time point). We then compared activation of the *HAC1* splicing reporter by tunicamycin and auxin-induced Alg13 degradation. Remarkably, Alg13 depletion activated the *HAC1* splicing reporter with similar kinetics and to a similar extent as treatment with 1 µg/ml tunicamycin, which is a standard condition for causing strong ER stress (Figure 6B; Travers *et al.*, 2000; Schuck *et al.*, 2009). Treatment with 2 µg/ml tunicamycin consistently yielded slightly stronger activation of the *HAC1* splicing reporter than treatment with 1 µg/ml tunicamycin or auxin. Whether this trend reflected less complete inhibition of protein N-glycosylation by 1 µg/ml tunicamycin and auxin or non-specific effects of treatment with 2 µg/ml tunicamycin is unclear. Activation of the UPR leads to expansion of the ER (Schuck *et al.*, 2009). Analysis of ER morphology by fluorescence microscopy revealed that Alg13 depletion caused ER expansion, similarly to the effects of 1 µg/ml tunicamycin (Figure 6C). Quantification of ER morphology furthermore showed that expansion was due to a proliferation of ER sheets in both cases (Figure 6D). Hence, Alg13 depletion activates the UPR and induces ER expansion essentially as efficiently as Alg7 inhibition by tunicamycin.

**FIGURE 7:**
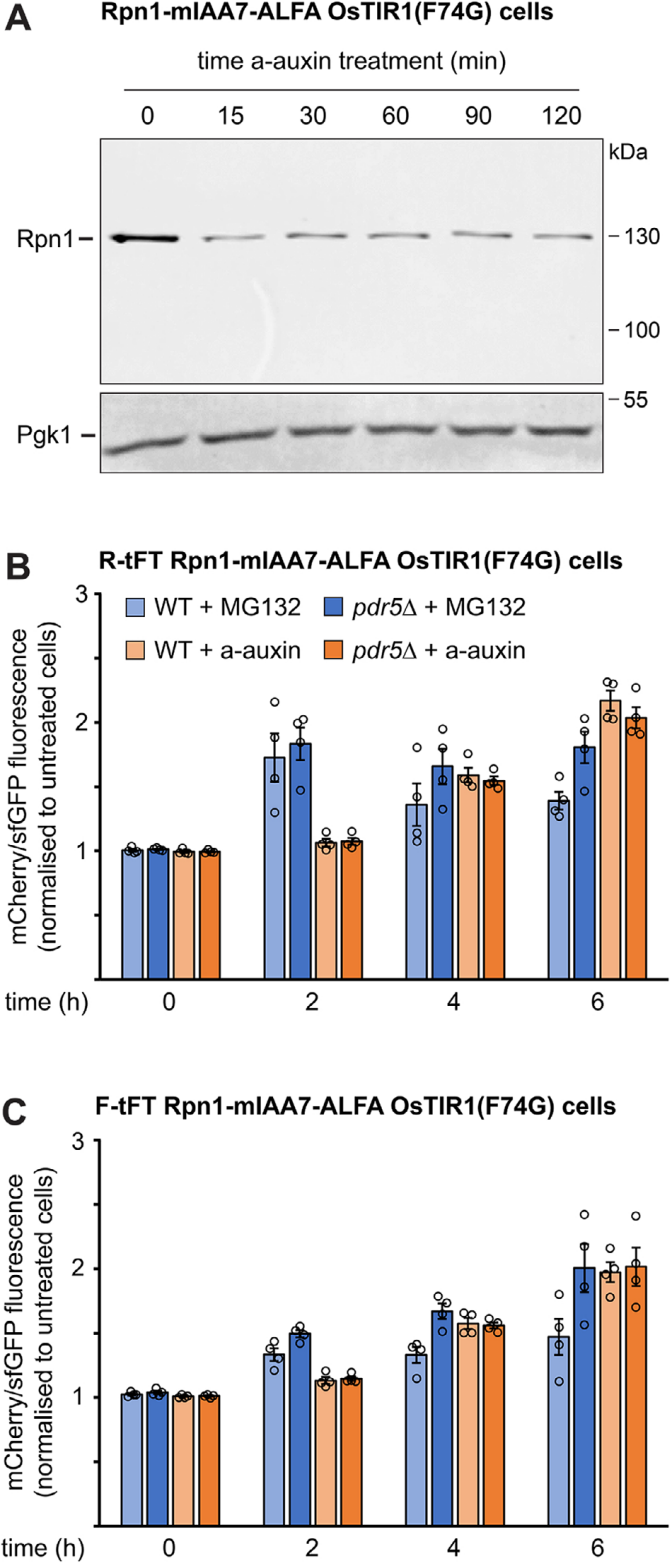
Inhibition of the proteasome by MG132 and auxin-induced Rpn1 depletion. (A) Western blot of ALFA tag from Rpn1-mIAA7-ALFA OsTIR1(F74G) cells after treatment with 0.5 µM a-auxin for the times indicated. Pgk1 served as a loading control. The experiment was done once. (B) Flow cytometry measurement of the mCherry/sfGFP fluorescence ratio of the R-tFT reporter in Rpn1-mIAA7-ALFA OsTIR1(F74G) cells that were otherwise wild-type (WT) or lacked the plasma membrane efflux pump Pdr5 (*pdr5*∆). Cells were left untreated or treated with 80 µM MG132 or 0.5 µM a-auxin. The mCherry/sfGFP fluorescence ratios in treated cells were normalized to the corresponding ratios in untreated cells. Bars show the mean of *n* = 4 biological replicates, error bars show the standard error of the mean. (C) As in panel B but with cells expressing the F-tFT reporter.

MG132 is a well-established proteasome inhibitor. MG132 has been reported to be ineffective in wild-type yeast but inhibits proteasomal protein degradation in mutants in which net uptake of MG132 into cells has been enhanced. This can be achieved by the removal of the ergosterol biosynthesis factor Erg6 or the plasma membrane efflux pump Pdr5 (Lee and Goldberg, 1996; Fleming *et al.*, 2002). Recent high-throughput data demonstrated that the auxin system can be used for proteasomal degradation of many proteasome subunits, that is, for self-degradation of the proteasome (Gameiro *et al.*, 2025; Valenti *et al.*, 2025). After some initial candidate testing, we settled on Rpn1 as the target proteasome subunit. Rpn1 belongs to the 20% most abundant proteins in yeast and is approximately as abundant as Sec63 (Platzek *et al.*, 2025). The levels of Rpn1 fused to mIAA7 and an ALFA tag (Götzke *et al.*, 2019) declined steeply within 15 min of auxin treatment but then remained constant, with substantial residual Rpn1 (Figure 7A). Hence, the new equilibrium levels of Rpn1, which are dictated by the combined rates of synthesis and auxin-dependent as well as auxin-independent degradation, were higher than those of Sec63 (refer to Figure 3E). It is possible that the degradation rate of Rpn1 was limited by a lack of functional proteasomes, which resulted from the initial drop in Rpn1 abundance.

To determine whether Rpn1 depletion was indeed sufficient to reduce proteasome activity, we employed tandem fluorescent timers, called R-tFT and F-tFT (Khmelinskii *et al.*, 2012). These constructs consisted of N-terminal arginine or phenylalanine residues as determinants for proteasomal degradation via the N-degron pathway, a linker sequence that can be ubiquitinated, a slow-maturing mCherry fluorescent protein, and a fast-maturing sfGFP fluorescent protein. Newly synthesized reporter molecules rapidly acquire green sfGFP fluorescence and, gradually and more slowly, red mCherry fluorescence. If a reporter is short-lived, it is degraded before maturation of mCherry is complete and shows a low ratio of mCherry to sfGFP fluorescence. Hence, the mCherry/sfGFP fluorescence ratio correlates with protein half-life and provides a readout for reporter stability. We introduced R-tFT and F-tFT reporters into wild-type and *pdr5∆* cells containing Rpn1-mIAA7-ALFA and OsTIR1(F74G), treated cells with MG132 or a-auxin, and measured the mCherry/sfGFP fluorescence ratio by flow cytometry for up to 6 h. Treatment with MG132 increased the fluorescence ratio and hence reporter half-life already in wild-type cells and more strongly in *pdr5∆* cells (Figure 7, B and C). For the R-tFT reporter, stabilization in *pdr5∆* cells was maximal after 2 h and then plateaued. For the F-tFT reporter, stabilization in *pdr5∆* cells was clearly apparent after 2 h and then further increased up to the 6 h time point. Treatment with a-auxin had little effect after 2 h but then progressively increased the stabilities of both reporters. After 6 h, the increase was comparable to or even somewhat stronger than that caused by MG132. Furthermore, auxin-induced reporter stabilization was equally strong in wild-type and *pdr5∆* cells. Hence, the auxin system can be applied in otherwise wild-type cells and achieves similar proteasome inhibition as MG132.

**FIGURE 8:**
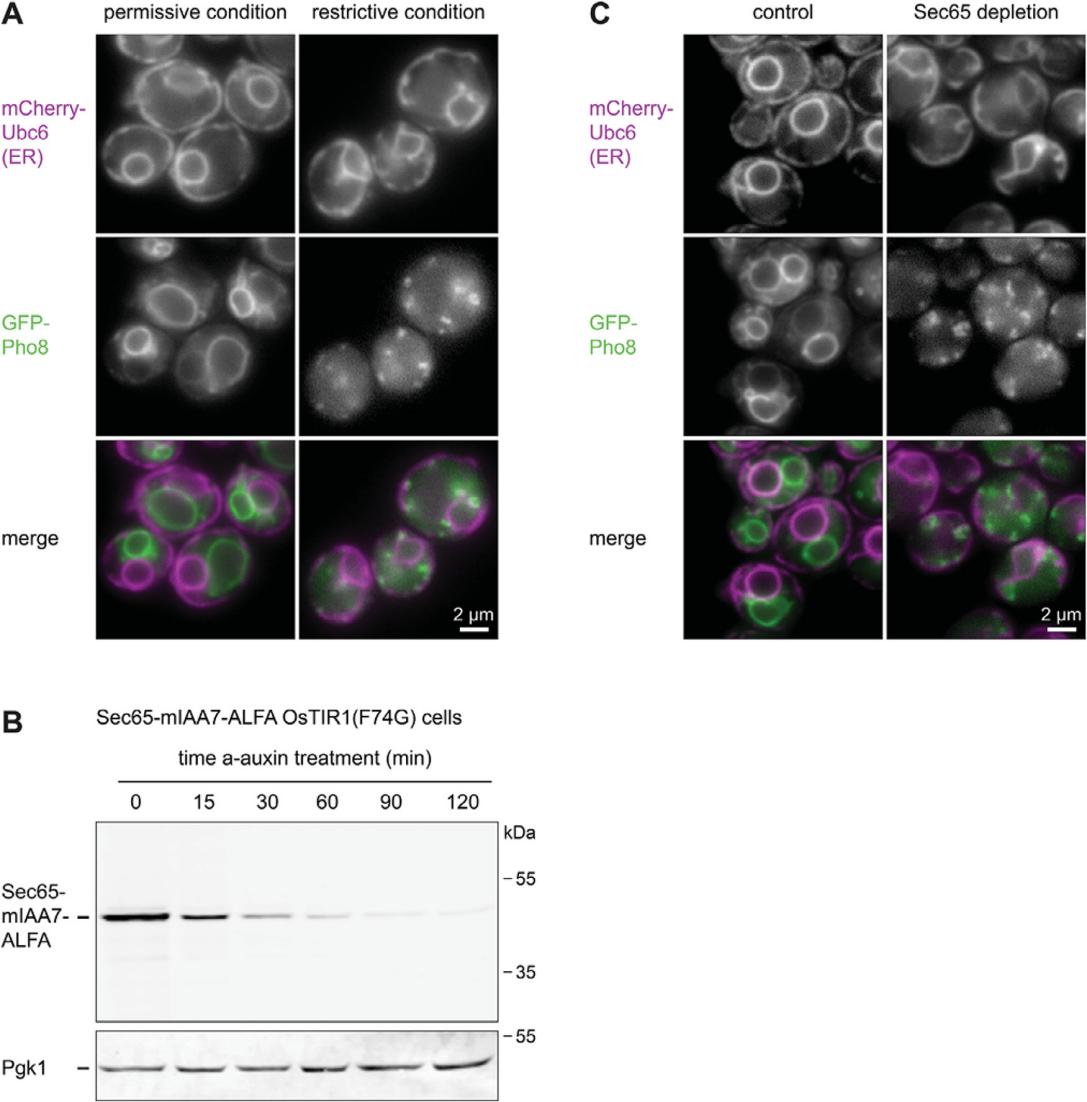
Inactivation of signal recognition particle by the *sec65-1* temperature-sensitive allele or auxin-induced Sec65 depletion. (A) Images of *sec65-1* cells constitutively expressing the general ER marker protein mCherry-Ubc6 and expressing the vacuole membrane protein GFP-Pho8 under the control of the estradiol-inducible system. Cells were either grown at 25°C and treated with estradiol at 25°C for 60 min (permissive condition) or grown at 25°C, shifted to 37°C for 60 min and treated with estradiol at 37°C for 60 min (restrictive condition). GFP-Pho8 fails to reach the vacuole membrane under restrictive conditions. (B) Western blot of ALFA tag from Sec65-mIAA7-ALFA OsTIR1(F74G) cells after treatment with 0.5 µM a-auxin for the times indicated. Pgk1 served as a loading control. The experiment was done once. (C) Images of Sec65-mIAA7-ALFA OsTIR1(F74G) cells constitutively expressing the general ER marker mCherry-Ubc6 and expressing the vacuole membrane protein GFP-Pho8 under the control of the estradiol-inducible system. Cells were either grown at 30°C and treated with estradiol for 60 min (control) or grown at 30°C, treated with 0.5 µM a-auxin for 60 min and additionally treated with estradiol for another 60 min (Sec65 depletion). The experiment was done twice. Sec65 depletion causes the same Pho8 transport and ER morphology defects observed in *sec65-1* cells at the restrictive temperature.

Finally, we tested whether the auxin system can be used in place of *ts* alleles. These mutant alleles are important tools to study gene function, especially of essential genes, which cannot be knocked out. Proteins encoded by *ts* alleles are at least partially functional at permissive temperatures but can be rendered non-functional by shifting cells to a different, typically higher, non-permissive temperature. As a test case, we used the established *sec65-1* allele. Sec65 is a subunit of the signal recognition particle and is essential for the co-translational translocation of many secretory proteins into the ER (Hann *et al.*, 1992). When *sec65-1* cells are shifted from the permissive temperature of 25°C to the restrictive temperature of 37°C, signal recognition particle is inactivated and ER insertion of certain newly synthesized proteins fails (Ng *et al.*, 1996). To reproduce this phenotype, we shifted wild-type and *sec65-1* cells from 25°C to 37°C for 60 min (Stirling *et al.*, 1992), then induced expression of the vacuolar transmembrane protein GFP-Pho8 with the *GEM-GAL* system for an additional 60 min, and finally imaged cells by fluorescence microscopy. As expected, newly synthesized GFP-Pho8 was inserted into the ER and transported to the vacuole membrane under the permissive condition (Figure 8A). By contrast, GFP-Pho8 was unable to reach the vacuole under the restrictive condition and accumulated in cytoplasmic puncta, possibly reflecting GFP-Pho8 aggregation in the cytosol or even mistargeting to fragmented mitochondria (Costa *et al.*, 2018). In addition, the ER structure was disturbed, consistent with earlier observations (Costa *et al.*, 2018). We then generated Sec65-mIAA7-ALFA cells and confirmed by Western blotting that auxin-induced degradation strongly diminished Sec65-mIAA7-ALFA levels within 60 min (Figure 8B). Next, we combined auxin-induced degradation of Sec65 for 60 min with subsequent estradiol-induced GFP-Pho8 expression for 60 min, all at the optimal growth temperature of 30°C. GFP-Pho8 was efficiently transported to the vacuole in the presence of Sec65 but was trapped in cytoplasmic puncta when Sec65 had been depleted. In addition, the ER structure was aberrant in Sec65-depleted cells (Figure 8C). Hence, the auxin system presents an alternative to *ts* alleles.

## DISCUSSION

In this study, we have systematically evaluated combinations of auxin-binding degrons, auxin derivatives and degron receptors. We found that the mIAA7 and AID* degrons together with a-auxin and OsTIR1(F74G) yield rapid and extensive target protein depletion, with little basal degradation in the absence of auxin. We showed that OsTIR1(F74G) should be expressed at high enough but not excessive levels and developed an inducible OsTIR1(F74G) expression system to tune its levels to target protein abundance. This system can replace chemical inhibitors, such as tunicamycin or MG132, and temperature-sensitive alleles. We have generated plasmids for chromosomal tagging of target proteins with various degron cassettes that include 3xFLAG, ALFA, mNeonGreen or mCherry tags for target protein analysis (Supplemental Table S3). Moreover, we have constructed various integrative plasmids for stable expression of degron receptor variants, including OsTIR1(F74G) under the control of constitutive or inducible promoters (Supplemental Table S4). All plasmids are available via the European Plasmid Repository and Addgene.

As shown here, and previously by others, the auxin system can target cytosolic proteins, membrane-associated proteins with cytosol-exposed degrons, and also nuclear proteins (Gameiro *et al.*, 2025; Valenti *et al.*, 2025). Furthermore, the system performs well for transmembrane proteins, which must be extracted from membranes before proteasomal degradation, and for proteins that are part of stable complexes, such as histones and proteasomes. The reach of the auxin system is limited for proteins localized entirely inside membrane-bound organelles because these proteins are inaccessible to the proteasome. Still, some depletion may be possible for newly synthesized organelle proteins that are targeted to their final destination post-translationally and can be attacked by the auxin system while they are still in transit. This appears to be the case for a number of mitochondrial proteins (Valenti *et al.*, 2025).

Target protein depletion by about 90% within 30 min was achieved for Sec63, Vph1, and Tom70, which are among the 10%–20% most abundant proteins in yeast. For proteins among the 5% most abundant proteins, depletion by >90% took longer, as for Tef4 and Htb1, or was not achievable, as for Fba1. We also tested Tdh3, which is the most abundant protein in our W303 strain background (Platzek *et al.*, 2025). Depletion was limited to 10% (unpublished data). This inverse correlation between target protein abundance and depletion efficiency has been noted before (Gameiro *et al.*, 2025; Valenti *et al.*, 2025). Highly abundant target proteins have a high synthesis rate in dividing yeast cells so that even high rates of auxin-induced degradation will result in a new steady state at which substantial residual amounts of the target protein are present. However, it remains unclear what factor limits auxin-induced degradation. We show that it is not the abundance of the degron receptor. In fact, excessive OsTIR1(F74G) can inhibit target protein degradation, as seen for expression driven by the extremely strong *GPD* promoter. An explanation for this counterintuitive effect may be that, at very high concentrations, OsTIR1(F74G) molecules no longer bridge auxin-bound degrons and ubiquitin ligases but separately saturate their respective binding sites at the target protein and the ubiquitin ligase, thus hindering recognition of target proteins by the ubiquitin-proteasome system.

The two degrons that worked well in our hands were AID*, which was recently used for the construction of genome-wide strain collections (Morawska and Ulrich, 2013; Gameiro *et al.*, 2025; Valenti *et al.*, 2025), and mIAA7 (Li *et al.*, 2019). mIAA7 is somewhat larger than AID* but was slightly superior regarding basal degradation, which is why we used it as our standard degron. We always fused target proteins to cassettes consisting of a degron and a tag so that auxin-induced degradation was easy to monitor. The presence of the additional tag may in some cases interfere with target protein function. However, tags could also stabilize the degron, especially when they contain stably folded protein domains, as is the case for fluorescent proteins. For instance, it has been reported that auxin-inducible degradation of the human seipin protein with mIAA7 alone was slow, was faster when the small 3xFLAG tag was appended to the degron and was even faster when a fluorescent protein was used as a tag (Li *et al.*, 2019). Hence, it may depend on the target protein whether the best choice is fusion with only a degron, a degron followed by a small epitope tag, or a degron followed by a folded domain. We have not investigated fusions of degrons to the N-termini of target proteins, but earlier work indicates that this approach is also possible, including for the mIAA7 degron (Nishimura *et al.*, 2009; Li *et al.*, 2019).

We show that the auxin system is competitive with chemical inhibitors and *ts* alleles. As long as fusion of a degron to a target protein is functionally neutral and a-auxin does not have unintended side effects, the auxin system may, in fact, offer considerable advantages over inhibitors and *ts* alleles. First, inhibitors can have off-target effects. For instance, tunicamycin causes ER stress but also impacts the mitochondrial proteome in a way that appears unrelated to ER stress (Platzek *et al.*, 2025). Thus, Alg13 depletion is a new and specific tool to induce ER stress. Second, the auxin system may sometimes be more potent or easier to apply. For instance, it is difficult to introduce MG132 into yeast cells at effective concentrations, even when Erg6 or Pdr5 has been removed, or special growth conditions are applied (Lee and Goldberg, 1996; Fleming *et al.*, 2002; Liu *et al.*, 2007). In addition, MG132 only inhibits the chymotryptic activity of the proteasome. Its tryptic and caspase-like activities remain, explaining why MG132 cannot block proteasome activity completely (Collins *et al.*, 2010; Howard *et al.*, 2012). Finally, MG132, which is a peptide aldehyde, appears to be unstable in yeast cells and needs to be replenished after some time (Lee and Goldberg, 1996). Our observation that auxin-induced depletion of Rpn1 in otherwise wild-type cells inhibited the proteasome at least as strongly as MG132 treatment in *pdr5∆* cells establishes an effective, simple-to-use new tool to study protein degradation in yeast. Rpn1 plays key roles in the delivery of ubiquitinated substrates to the proteasome (Shi *et al.*, 2016). It remains to be determined whether Rpn1 depletion affects all proteasome substrates and whether depletion of another proteasome subunit would provide even tighter proteasome inhibition. Third, *ts* alleles require experimental set-ups in which yeast cells are shifted from a permissive to a restrictive temperature, neither of which may be optimal for cell physiology. In addition, it needs to be ascertained that any phenotype results from the inactivation of the mutant protein and not from the temperature shift itself. The auxin system can, in principle, be used at any growth temperature. Fourth, the auxin system requires no target-specific reagents and only involves standard genome manipulation. By contrast, the development of new chemical inhibitors and *ts* alleles cannot be standardized as easily, requires more work, or both.

Considering the efficiency of the auxin system, its application will likely become even more widespread. The reagents now readily available, including genome-wide collections of degron strains (Gameiro *et al.*, 2025; Valenti *et al.*, 2025) and the set of plasmids described here, make it easy for researchers to adopt the auxin system as a standard approach to investigate protein function in yeast. We expect that the consistent use of auxin-inducible protein degradation will alleviate problems caused by cell adaptation or off-target effects and enable new biological insight.

## MATERIALS AND METHODS

Request a protocol through *Bio-protocol*

### Plasmids

Plasmids used in this study are listed in Supplemental Table S1. Plasmids generated in this study as tools for the auxin system (Supplemental Tables S3 and S4) are available from the European Plasmid Repository (www.plasmids.eu) and Addgene (www.addgene.org).

Plasmids of the pFA6a series have been described (Longtine *et al.*, 1998; Janke *et al.*, 2004; Papagiannidis *et al.*, 2021). The new kanNP1 module consists of the *S. cerevisiae TPI1* promoter, the *E. coli* transposon *Tn903* kanR open reading frame and the *A. gossypii TEF* terminator. It replaces the kanMX6 module and avoids unwanted recombination in yeast containing both the kanMX6 and the HIS3MX6 module. mAID, AID*, and mIAA7 sequences (Yesbolatova *et al.*, 2020; Morawska and Ulrich, 2013; Li *et al.*, 2019) were inserted into pFA6a plasmids as synthetic gene fragments by Gibson assembly cloning. The OsTIR1(F74G) sequence was amplified from P_GAL_-OsTIR1(F74G)-URA3 (Yesbolatova *et al.*, 2020) and inserted into pRS405/6-P_ADH_ (Mumberg *et al.*, 1995). The P_ADH_-OsTIR1(F74G) cassette was then inserted into pNH604/5 vector backbones (Pincus *et al.*, 2014). In the process, the *ADH* promoter was shortened to approximately 700 bp and thus slightly weakened, as in earlier pNH604/5 plasmids (Pincus *et al.*, 2014). Similarly, the AtAFB2 sequence from pSH-EFIRES-B-AtAFB2-mCherry (Li *et al.*, 2019) was inserted into pRS405-P_ADH_, and the P_ADH_-AtAFB2 cassette with the shortened *ADH* promoter was then inserted into the pNH605 vector backbone. Site-directed mutagenesis was used to generate the variants OsTIR1, OsTIR1(F74A), OsTIR1(F74G,S210A), AtAFB2(F74A) and AtAFB2(F74G). To generate pRS303K-P_GPD_-sfGFP, pRS303K-P_GPD_-TagBFP (Szoradi *et al.*, 2018) was linearized by PCR and combined with the sfGFP sequence by Gibson assembly cloning, replacing TagBFP. To generate pRS303K-P_RNR1_-sfGFP and pRS303K-P_ADH_-sfGFP, the *RNR1* and the shortened *ADH* promoter were amplified from pYTK021 (Lee *et al.*, 2015) and pNH604-P_ADH_-OsTIR1(F74G), respectively, and inserted into pRS303K-P_GPD_-sfGFP, replacing the *GPD* promoter. To generate pRS303K-P_XXX_-OsTIR1(F74G) plasmids, the OsTIR1(F74G) sequence was amplified from pNH604-P_ADH_-OsTIR1(F74G) and inserted into pRS303K-P_GPD_-sfGFP, pRS303K-P_ADH_-sfGFP and pRS303K-P_RNR1_-sfGFP, replacing sfGFP. pRS303H/N-OsTIR1(F74G) were generated by insertion of OsTIR1(F74G) into pRS303H/N (Taxis and Knop, 2006). pRS303K-P_GAL_-sfGFP was generated by replacing the *RNR1* promoter in pRS303K-P_RNR1_-sfGFP with the *GAL* promoter from pNH605-P_ADH_-GEM-P_GAL_ (Schmidt *et al.*, 2019). Next, the P_ADH_-GAL4-ER-Msn2 (GEM) sequence from pNH605-P_ADH_-GEM-P_GAL_ was inserted, yielding pRS303K-P_GAL_-sfGFP-P_ADH_-GEM. Finally, sfGFP was replaced by the OsTIR1(F74G) sequence, yielding pRS303K-P_GAL_-OsTIR1(F74G)-P_ADH_-GEM.

### Yeast strains

Strains in this study were in the W303 background (genotype *ADE2 leu2-3,112 trp1-1 ura3-1 his3-11,15 MATa*) and are listed in Supplemental Table S2. Gene tagging and deletion were done with PCR products (Longtine *et al.*, 1998; Janke *et al.*, 2004). Supplemental Table S3 lists the primers to be used with each template plasmid for gene tagging. For a given gene, primers R1 and S2 can be used interchangeably but primers F2 and S3 cannot. Even though all pFA6a plasmids have sequences complementary to the F2 and S3 primers, each pFA6a plasmid yields a PCR product with the correct reading frame with only one of them. Expression plasmids for the degron receptors were digested with restriction enzymes before integration into the genome. Supplemental Table S4 lists the restriction enzymes that can be used for this purpose. Note that multiple copies of linearized pRS405/6 plasmids can integrate into the genome, whereas pNH604/5 and pRS303H/K/N are single-integration plasmids.

### Growth assays

Strains were grown in liquid culture at 30°C in synthetic complete (SC) medium containing 0.7% yeast nitrogen base without amino acids (Merck), amino acids and 2% glucose. Precultures were inoculated in 1 ml medium in 96-deep well plates and grown for 24 h so that they reached saturation. Precultures were diluted 1:1000 into 1 ml fresh medium, and cultures were grown at 30°C for 9 h and the optical density of the cultures at 620 nm (OD_620_) was measured with a plate reader Infinite F50 Plus (Tecan).

### Flow cytometry

Precultures were grown to saturation as described above, diluted into 1 ml fresh medium and grown overnight so that they reached mid-log phase (OD_600_ = 0.1 – 0.5). Overnight cultures were diluted to OD_600_ = 0.05 and then either not treated or treated with 375 µM indole-3-acetic acid (n-auxin, Sigma-Aldrich), 5 µM 5-phenyl-indole-3-acetic acid (p-auxin, Sigma-Aldrich), 0.5 µM 5-adamantyl-indole-3-acetic acid (a-auxin, TCI Chemicals), 800 nM estradiol (Sigma-Aldrich), 1 or 2 µg/ml tunicamycin (Sigma-Aldrich), or 80 µM MG132 (Thermo Fisher Scientific) for the times indicated. Total cell fluorescence after excitation with a 488 nm laser was measured with a FACSCanto or a FACSymphony A1 flow cytometer (BD Biosciences), and the geometric mean of the cell population was calculated with FlowJo software. Cell autofluorescence was determined with a strain not expressing a fluorescent protein and subtracted from all other measurements. Cellular protein levels were determined by dividing the background-corrected cell fluorescence by the forward scatter, which served as a measure for cell size.

To analyze UPR activation with the *HAC1* splicing reporter, cells expressing cytosolic BFP as an indicator for cellular protein translation capacity were used. Background-corrected GFP fluorescence was divided by background-corrected BFP fluorescence elicited with a 405 nm laser, and the GFP/BFP ratio in treated cells was normalized to the ratio in untreated cells, yielding fold induction of the reporter upon treatment.

To analyze proteasome activity, mCherry and sfGFP fluorescence were measured in cells expressing R-tFT or F-tFT reporters after excitation with a 561 nm and a 488 nm laser, respectively. Values were corrected for autofluorescence by subtracting values for mCherry and sfGFP fluorescence determined in cells not expressing a tFT reporter, and the ratio of background-corrected mCherry/sfGFP fluorescence was calculated. The mCherry/sfGFP ratio in treated cells was normalized to the ratio in untreated cells.

### Western blotting

Strains were grown to mid-log phase in liquid culture at 30°C in SC medium. Cultures were diluted to OD_600_ = 0.3 and treated with 0.5 µM a-auxin for up to 2 h. At each time point, five ODs of cells were collected by centrifugation, washed with water, resuspended in lysis buffer (50 mM HEPES pH 7.5, 0.5 mM EDTA, Roche complete protease inhibitors), and disrupted by bead beating with a FastPrep 24 (MP Biomedicals). Proteins were solubilized by the addition of 1.5% (wt/vol) SDS and incubation at 65°C for 5 min. Lysates were cleared at 16,000 g at 4°C for 2 min and protein concentrations were determined with a BCA kit (Pierce). A total of 20 µg total cell protein was separated by Tris-glycine SDS–PAGE and transferred onto PVDF membranes by semidry blotting. Membranes were probed with primary and fluorophore-coupled secondary antibodies. For fluorescence detection, membranes were analyzed with an Odyssey CLx imaging system and Image Studio software (LI-COR Biosciences). Antibodies were rabbit anti-OsTIR1 (MBL Life Science, RRID:AB_2909494), mouse anti-FLAG (Merck, RRID:AB_262044), mouse anti-Pgk1 (Abcam, RRID:AB_10861977), single-domain anti-ALFA coupled to IRDye 800CW (NanoTag Biotechnologies, RRID:AB_3075985), donkey anti-rabbit IRDye 800CW (LI-COR, RRID: AB_621848), goat anti-mouse IRDye 800CW (LI-COR, RRID:AB_621842), and goat anti-mouse Alexa-680 (Invitrogen, RRID:AB_141436).

### Microscopy

To image ER remodeling upon ER stress or Alg13 depletion, strains were grown to mid-log phase in liquid SC medium at 30°C. Cultures were diluted to OD_600_ = 0.2 and either not treated or treated with 1 µg/ml tunicamycin or 0.5 µM a-auxin for 2 h. Cells from 1 ml of culture were collected by centrifugation and resuspended in 20 µl SC. In total, 3 µl of cell suspension were applied to a glass coverslip and covered with a 1% agarose pad prepared with SC medium. Images were acquired with a DMi8 inverted microscope (Leica) equipped with a CSU-X1 spinning-disk confocal scanning unit (Yokogawa) and an ORCA-Flash 4.0 LT camera (Hamamatsu). A HC PL APO 63x/1.40-0.60 oil objective lens (Leica) was used. For image analysis, images were anonymized with the “Blind Analysis Tools” plugin in ImageJ (https://imagej.net/plugins/blind-analysis-tools) to prevent user bias. Cells were then segmented using CellX software (Dimopoulos *et al.*, 2014), and ER morphology (% cell cortex coverage, ER tubules and ER sheets) was quantified with the ClassifiER script in MATLAB (Papagiannidis *et al.*, 2021). Twelve fields of view with at least 40 cells each were quantified per condition, and a 95% confidence interval was calculated based on the variance between fields of view.

To image ER translocation and transport of newly synthesized GFP-Pho8, strains were grown to mid-log phase in liquid SC medium at 25°C (*sec65-1* strain) or 30°C (Sec65-mIAA7-ALFA strain). Cultures of *sec65-1* cells were split in two, maintained at 25°C or shifted to 37°C for 60 min, treated with 800 nM estradiol to induce GFP-Pho8 expression for 60 min and imaged with a Nikon Ti2 widefield microscope equipped with a Nikon Plan Apo 100x/NA 1.45 objective and a Hamamatsu Orca Fusion-BT camera. Cultures of Sec65-mIAA7-ALFA cells were split into two, not treated or treated with 0.5 µM a-auxin for 60 min, and then treated with estradiol and imaged as above.

### Statistical tests

To test statistical significance of differences between two groups, a two-tailed unpaired heteroscedastic Student's *t* test was used when one group was normalized to the other and, therefore, the data of the reference group had no variance (basal degradation of Sec63 in Figure 1C; Tef4-mAID, Htb1-mAID and Sec63-mAID in Figure 1E; Figure 4A; Figure 5A). When the data of both groups had a variance, a two-tailed paired homoscedastic Student's *t* test was used. In some cases, such a paired test was done because the variation between biological replicates was of similar or greater magnitude than the variation between groups, which would have masked reproducible trends if an unpaired test had been used (Supplemental Figure S3, A–D). In other cases, the two groups each contained subsets of data that could be compared meaningfully only to the corresponding subset in the other group, which also necessitated a paired *t* test (datasets for OsTIR1(F74G) and AtAFB2(F74A) containing values for different target proteins in Supplemental Figure S4, G and H). To test statistical significance of differences between three groups, a one-way ANOVA followed by a Tukey test was used (OsTIR1, OsTIR1(F74G) and AtAFB2(F74A) in Supplemental Figure S1B; n-auxin, p-auxin, and a-auxin in Figure 2 and Supplemental Figure S2; mAID-, AID*- and mIAA7-tagged proteins in Figure 3 and Supplemental Figure S4).

## Supporting information





## Abbreviations used:

ER: endoplasmic reticulum
IAA: indole-3-acetic acid
mAID: mini-auxin inducible degron
mIAA7: mini-IAA7
sfGFP: super-folder GFP
tFT: tandem fluorescent timer
ts: temperature-sensitive
UPR: unfolded protein response.
