# Supplementary material for: Systematic evaluation of tools for auxin-inducible protein degradation in budding yeast
Source: Mol Biol Cell. 2025 Dec 16;37(2):mr2. doi: 10.1091/mbc.E25-12-0571 (PMC12879018; doi:10.1091/mbc.E25-12-0571)
Supplement: Supplementary file 1 [file mbc-37-mr2-s001.pdf]

Supplemental Materials

Molecular Biology of the Cell

Hubbe *et al*.

Systematic evaluation of tools for auxin-inducible protein degradation in budding yeast

Petra Hubbe, Charu Sharma, Oliver Pajonk, Niklas Peters, Nadja Guschtschin-Schmidt, Natalie Friemel, Sebastian Schuck

		page
**Figure S1.**	Basal degradation by auxin in the absence of a degron receptor	2
**Figure S2.**	Inducible protein degradation by n-/p-/a-auxin in the presence of AtAFB2(F74A)	4
**Figure S3.**	Inducible degradation by different auxins and degron receptor variants	5
**Figure S4.**	Kinetics of inducible protein degradation by a-auxin in the presence of OsTIR1(F74G) or AtAFB2(F74A)	7
**Figure S5.**	Strength of constitutive and inducible promoters for OsTIR1(F74G) expression	9
**Table S1.**	Plasmids used in this study	10
**Table S2.**	Yeast strains used in this study	12
**Table S3.**	Plasmids and cognate forward and reverse primers for gene tagging	15
**Table S4.**	Integrative expression plasmids for degron receptors	16

Figure S1

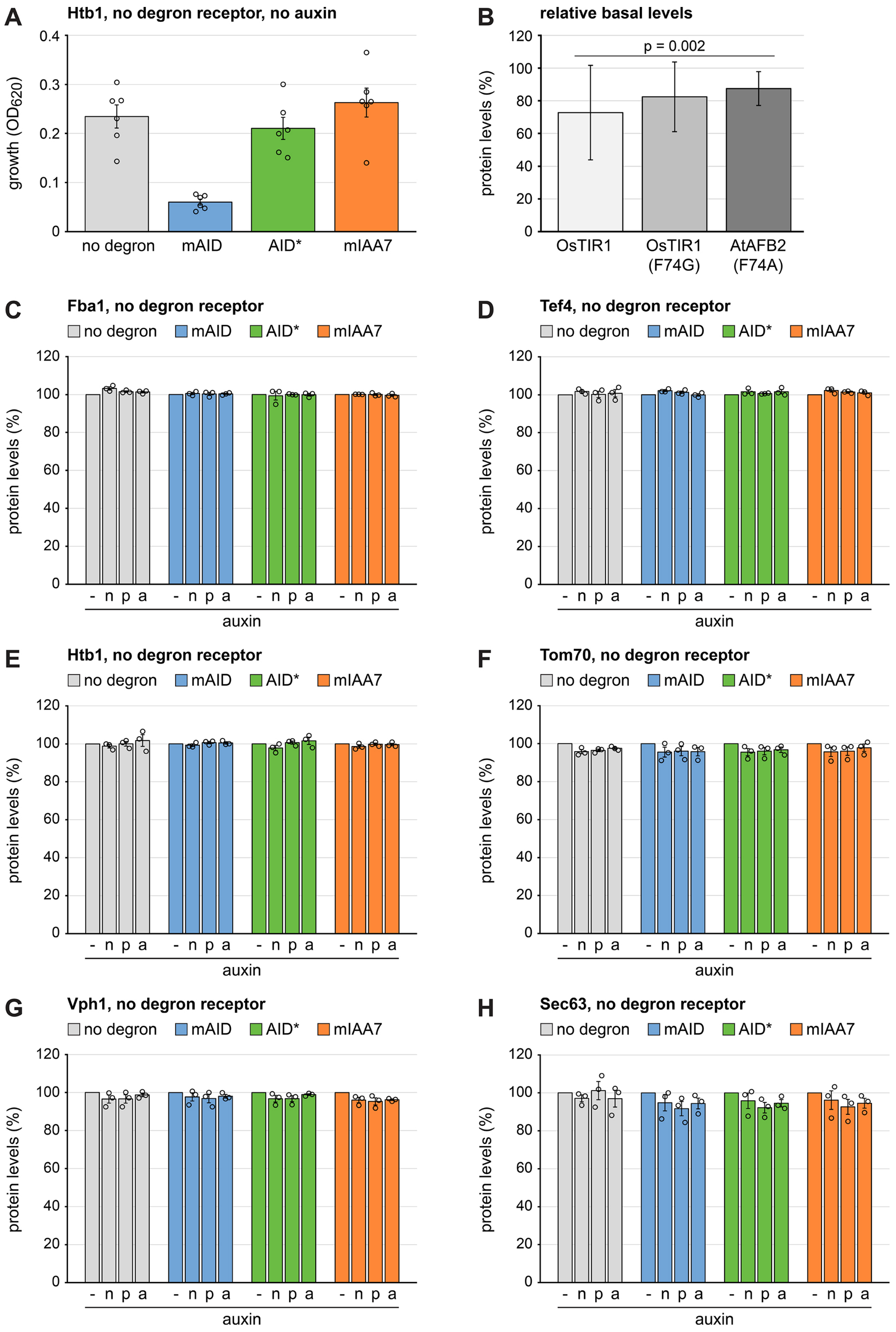
A
Htb1, no degron receptor, no auxin
growth (OD620)
0.4
0.3
0.2
0.1
0
no degron
mAID
AID*
mIAA7
B
relative basal levels
p = 0.002
protein levels (%)
120
100
80
60
40
20
0
OsTIR1
OsTIR1 (F74G)
AtAFB2 (F74A)
C
Fba1, no degron receptor
D
Tef4, no degron receptor
E
Htb1, no degron receptor
F
Tom70, no degron receptor
G
Vph1, no degron receptor
H
Sec63, no degron receptor
no degron
mAID
AID*
mIAA7
protein levels (%)
- n p a
auxin

Figure S1. Basal degradation by auxin in the absence of a degron receptor. **(A)** Results of growth assays showing the optical densities at 620 nm (OD_{620}) reached by cultures of the indicated strains at the end of a 9-hour growth period. Bars show the mean OD_{600} of n = 6 biological replicates, error bars show the standard error of the mean. **(B)** Basal levels of degron-tagged target proteins in presence of OsTIR1, OsTIR1(F74G) or AtAFB2(F74A) relative to the levels of proteins tagged only with mNeonGreen. Bars show the mean protein levels of all six target proteins tagged with mAID-, AID*- or mIAA7-mNeonGreen from n = 3 biological replicates, error bars show the standard deviation. Statistical significance of the difference between groups was evaluated with a one-way ANOVA. A subsequent Tukey test showed that the difference between OsTIR1 and AtAFB2(F74A) was significant at the 1% level. **(C)** Flow cytometry measurement of relative levels of Fba1 tagged with mNeonGreen (no degron), mAID-mNeonGreen (mAID), AID*-mNeonGreen (AID*) or mIAA7-mNeonGreen (mIAA7). Cells did not express a degron receptor and were either not treated or treated with 375 μM n-auxin, 5 μM p-auxin or 0.5 μM a-auxin for 120 minutes. Bars show the mean protein levels of n = 3 biological replicates, error bars show the standard error of the mean. Protein levels were normalized to the levels in untreated cells. **(D-H)** As in panel C but for Tef4, Htb1, Tom70, Vph1 and Sec63, respectively.

Figure S2

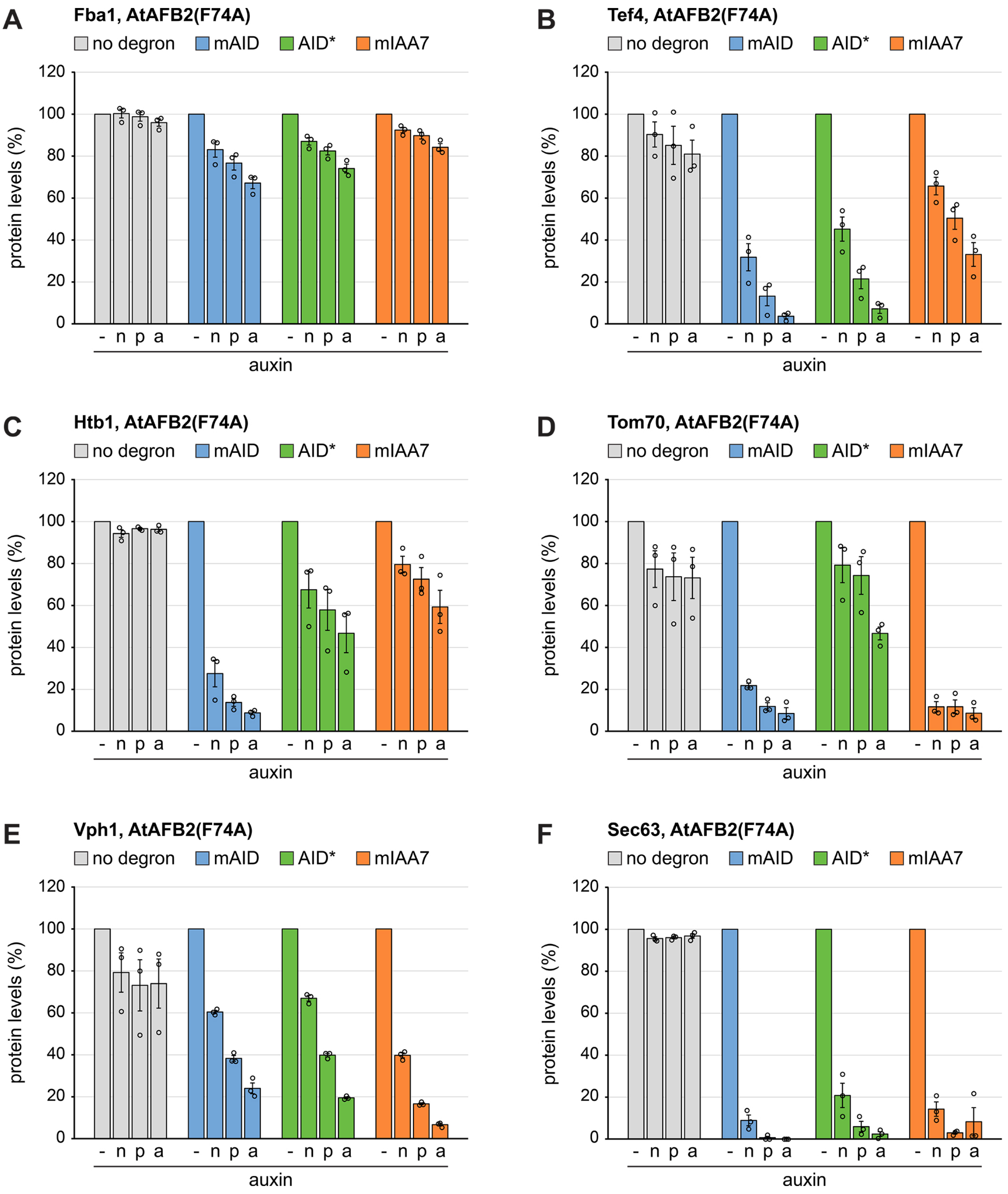

Figure S2. Inducible protein degradation by n-/p-/a-auxin in the presence of AtAFB2(F74A). **(A)** Flow cytometry measurement of relative levels of Fba1 tagged with mNeonGreen (no degron), mAID-mNeonGreen (mAID), AID*-mNeonGreen (AID*) or mIAA7-mNeonGreen (mIAA7). Cells expressed AtAFB2(F74A) and were not treated or treated with 375 μM n-auxin, 5 μM p-auxin or 0.5 μM a-auxin for 120 minutes. Bars show the mean protein levels of n = 3 biological replicates, error bars show the standard error of the mean. Protein levels were normalized to the levels in untreated cells. **(B-F)** As in panel A but for Tef4, Htb1, Tom70, Vph1 and Sec63, respectively.

Figure S3

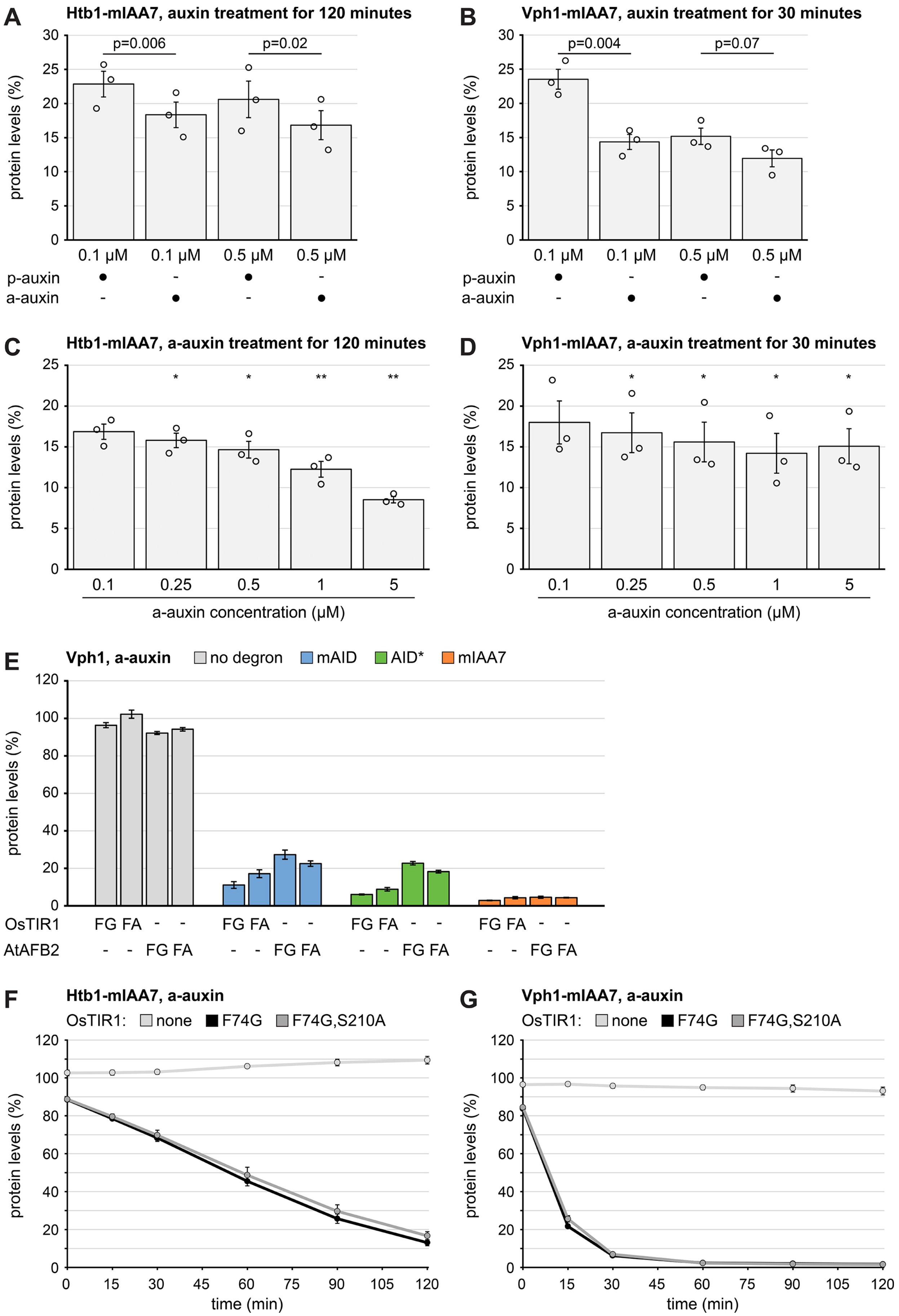

A
Htb1-mIAA7, auxin treatment for 120 minutes
p=0.006
p=0.02
protein levels (%)
0.1 µM
0.1 µM
0.5 µM
0.5 µM
p-auxin
a-auxin
B
Vph1-mIAA7, auxin treatment for 30 minutes
p=0.004
p=0.07
C
Htb1-mIAA7, a-auxin treatment for 120 minutes
a-auxin concentration (µM)
D
Vph1-mIAA7, a-auxin treatment for 30 minutes
E
Vph1, a-auxin
no degron
mAID
AID*
mIAA7
OsTIR1
AtAFB2
FG FA - -
- - FG FA
F
Htb1-mIAA7, a-auxin
OsTIR1:
none
F74G
F74G,S210A
time (min)
G
Vph1-mIAA7, a-auxin

Figure S3. Inducible degradation by different auxins and degron receptor variants. **(A)** Flow cytometry measurement of relative levels of Htb1-mIAA7-mNeonGreen (Htb1-mIAA7) after treatment with 0.1 or 0.5 μM p-auxin or a-auxin for 120 minutes. Bars show the mean protein levels of n = 3 biological replicates, error bars show the standard error of the mean. Protein levels were normalized to the levels in untreated cells. Statistical significance of the differences between p-auxin and a-auxin was determined with a two-tailed paired t-test. **(B)** As in panel A but for cells that expressed Vph1-mIAA7-mNeonGreen (Vph1-mIAA7) and were treated with auxin for 30 min. **(C)** Flow cytometry measurement of relative levels of Htb1-mIAA7-mNeonGreen (Htb1-mIAA7) after treatment with 0.1 - 5 μM a-auxin for 120 minutes. This time point showed the largest differences between different a-auxin concentrations. Bars show the mean protein levels of n = 3 biological replicates, error bars show the standard error of the mean. Protein levels were normalized to the levels in untreated cells. Statistical significance of the differences to the protein levels in cells treated with 0.1 μM a-auxin was evaluated with a two-tailed paired t-test. * $p<0.05$, ** $p<0.01$. **(D)** As in panel C but for cells that expressed Vph1-mIAA7-mNeonGreen (Vph1-mIAA7) and were treated with auxin for 30 min. This time point showed the largest differences between different a-auxin concentrations. **(E)** Flow cytometry measurement of relative levels of Vph1 tagged with mNeonGreen (no degron), mAID-mNeonGreen (mAID), AID*-mNeonGreen (AID*) or mIAA7-mNeonGreen (mIAA7). Cells expressed OsTIR1(F74G), OsTIR1(F74A), AtAFB2(F74G) or AtAFB2(F74A) and were either not treated or treated 0.5 μM a-auxin for 120 minutes. Bars show the mean protein levels in treated relative to untreated cells of n = 3 biological replicates, error bars show the standard error of the mean. OsTIR1(F74G) is slightly superior to OsTIR1(F74A), and AtAFB2(F74A) is slightly superior to AtAFB2(F74G). **(F)** Flow cytometry measurement of relative levels of Htb1-mIAA7-mNeonGreen (Htb1-mIAA7) in cells expressing either no OsTIR1, OsTIR1(F74G) or OsTIR1(F74G,S210A). Cells were treated with a-auxin for 0, 15, 30, 60, 90 or 120 minutes. Lines show the mean protein levels of n = 3 biological replicates, error bars show the standard error of the mean. Protein levels were normalized to the levels in cells that expressed Htb1-mNeonGreen and were treated with a-auxin for 0 min. Expression of the OsTIR1 variants causes approximately 10% basal degradation of Htb1-mIAA7-mNeonGreen. OsTIR1(F74G) mediates slightly faster auxin-induced degradation than OsTIR1(F74G,S210A). **(G)** As in panel F but for Vph1. Basal degradation by the OsTIR1 variants is approximately 15% and auxin-induced degradation is nearly identical for both OsTIR1 variants.

Figure S4

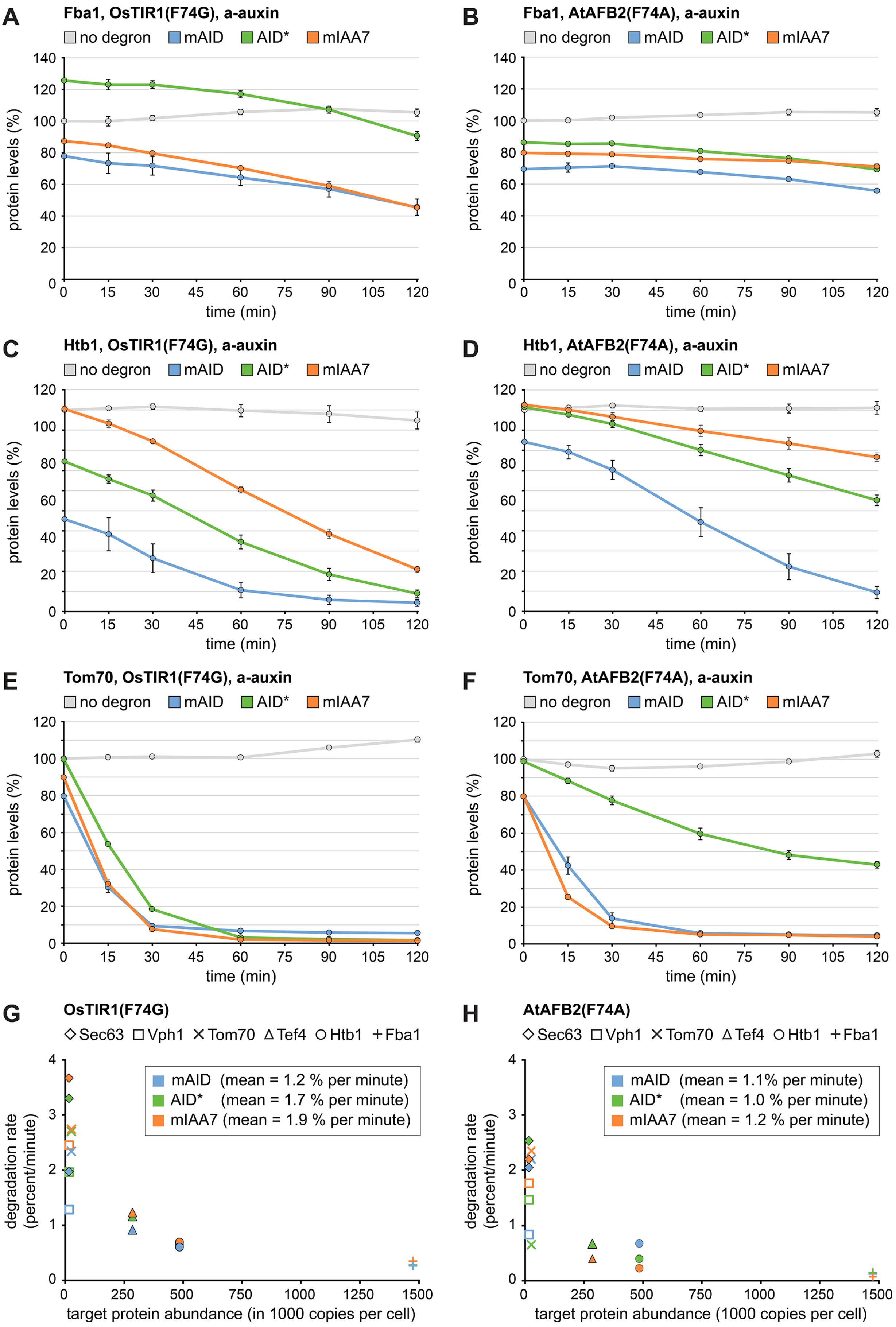

A
Fba1, OsTIR1(F74G), a-auxin
no degron
mAID
AID*
mIAA7
protein levels (%)
time (min)
B
Fba1, AtAFB2(F74A), a-auxin
C
Htb1, OsTIR1(F74G), a-auxin
D
Htb1, AtAFB2(F74A), a-auxin
E
Tom70, OsTIR1(F74G), a-auxin
F
Tom70, AtAFB2(F74A), a-auxin
G
OsTIR1(F74G)
Sec63
Vph1
Tom70
Tef4
Htb1
Fba1
mAID (mean = 1.2 % per minute)
AID* (mean = 1.7 % per minute)
mIAA7 (mean = 1.9 % per minute)
degradation rate (percent/minute)
target protein abundance (in 1000 copies per cell)
H
AtAFB2(F74A)
mAID (mean = 1.1% per minute)
AID* (mean = 1.0 % per minute)
mIAA7 (mean = 1.2 % per minute)
target protein abundance (1000 copies per cell)

Figure S4. Kinetics of inducible protein degradation by a-auxin in the presence of OsTIR1(F74G) or AtAFB2(F74A). **(A)** Flow cytometry measurement of relative levels of Fba1 tagged with mNeonGreen (no degron), mAID-mNeonGreen (mAID), AID*-mNeonGreen (AID*) or mIAA7-mNeonGreen (mIAA7). Cells expressed OsTIR1(F74G) and were treated with 0.5 μM a-auxin for 0, 15, 30, 60, 90 or 120 minutes. Lines show the mean protein levels of n = 3 biological replicates, error bars show the standard error of the mean. Protein levels were normalized to the levels in cells that expressed Tef4-mNeonGreen (no degron) and were treated with a-auxin for 0 min. **(B)** As in panel A but for cells expressing AtAFB2(F74A). **(C, E)** As in panel A but for cells expressing tagged variants of Htb1 and Tom70, respectively. **(D, F)** As in panel A but for cells expressing AtAFB2(F74A) and tagged variants of Htb1 and Tom70, respectively. **(G)** Degradation rates in cells expressing OsTIR1(F74G) given as percent protein levels per minute and derived by linear regression from the initial time points of the plots shown in Figures 3A, 3C, 3E, S4A, S4C and S4E. Degradation rates are plotted against target protein abundance taken from Platzek et al., 2025. Rates decline with increasing target protein abundance. **(H)** As in panel G but for cells expressing AtAFB2(F74A) and derived from the plots shown in Figures 3B, 3D, 3F, S4B, S4D and S4F. Degradation rates are overall lower than for cells expressing OsTIR1(F74G).

Figure S5

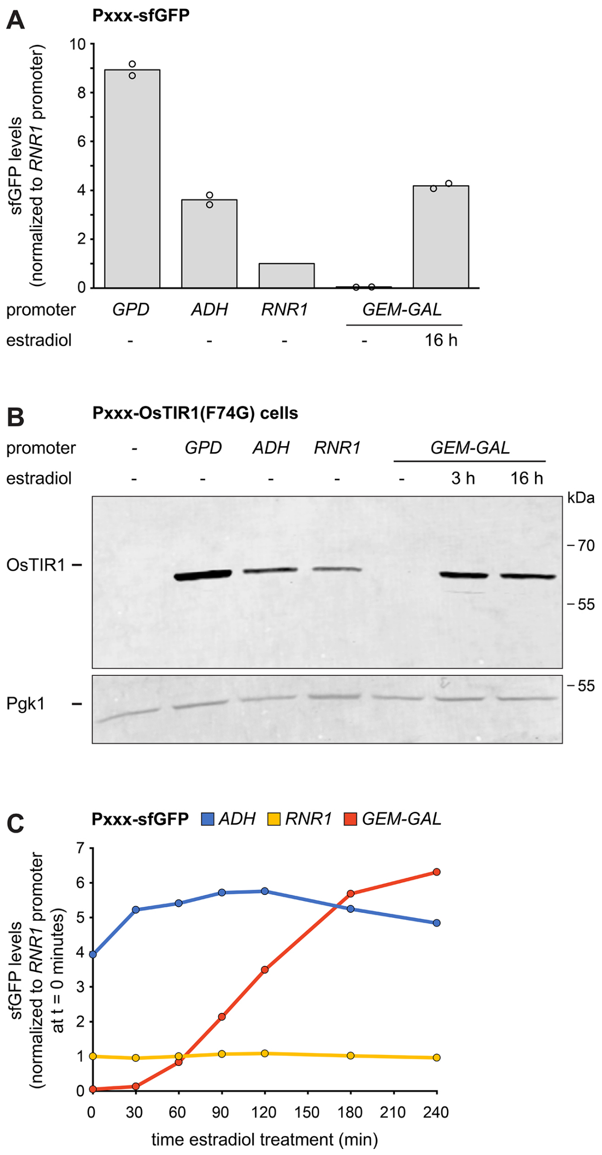

Figure S5. Strength of constitutive and inducible promoters for OsTIR1(F74G) expression. **(A)** Flow cytometry measurement of sfGFP levels in cells expressing sfGFP under the control of different constitutive promoters (Pxxx), i.e. the very strong *GPD* promoter, the moderate *ADH* promoter or the weak *RNR1* promoter, or the control of the inducible *GEM-GAL* system. Cells were grown in the absence of estradiol or treated with 800 nM estradiol for 16 hours. Bars show the mean of n = 2 technical replicates. sfGFP levels were normalized to the levels in cells expressing sfGFP under the control of the *RNR1* promoter. **(B)** Western blot of OsTIR1(F74G) expressed under the control of different constitutive promoters (Pxxx) or the *GEM-GAL* system. Where indicated, cells were treated with 800 nM estradiol for 3 or 16 hours. Pgk1 served as a loading control. The experiment was done once. **(C)** As in panel A and showing sfGFP levels over time after treatment with 800 nM estradiol for up to 4 hours. Lines show the mean of n = 2 technical replicates. sfGFP levels were normalized to the levels in cells expressing sfGFP under the control of the *RNR1* promoter at t = 0 minutes.

Table S1. Plasmids used in this study. GEM, GAL4-ER-Msn2; sfGFP, superfolder GFP.

Plasmid	Alias	Source
pFA6a-mAID-mNeonGreen-HIS3MX6	pSS1322	this study
pFA6a-mAID-mNeonGreen-klTRP1	pSS1323	this study
pFA6a-AID*-mNeonGreen-HIS3MX6	pSS1483	this study
pFA6a-mIAA7-mNeonGreen-HIS3MX6	pSS1392	this study
pFA6a-mIAA7-mNeonGreen-klTRP1	pSS1393	this study
pFA6a-mIAA7-mNeonGreen-kanNP1	pSS1637	this study
pFA6a-mIAA7-mNeonGreen-natNT2	pSS1639	this study
pFA6a-mIAA7-mNeonGreen-hphNT1	pSS1638	this study
pFA6a-mIAA7-mCherry-HIS3MX6	pSS1626	this study
pFA6a-mIAA7-mCherry-klTRP1	pSS1625	this study
pFA6a-mIAA7-mCherry-kanNP1	pSS1617	this study
pFA6a-mIAA7-3xFLAG-HIS3MX6	pSS1475	this study
pFA6a-mIAA7-3xFLAG-klTRP1	pSS1476	this study
pFA6a-mIAA7-3xFLAG-kanNP1	pSS1616	this study
pFA6a-mIAA7-ALFA-HIS3MX6	pSS1633	this study
pFA6a-mIAA7-ALFA-klTRP1	pSS1634	this study
pFA6a-mIAA7-ALFA-kanNP1	pSS1635	this study
pGAL-OsTIR1(F74G)-URA3	pSS1217	Yesbolatova et al., 2020
pRS405-P_{ADH}-OsTIR1(F74G)	pSS1330	this study
pRS406-P_{ADH}-OsTIR1(F74G)	pSS1331	this study
pNH605-P_{ADH}-OsTIR1(F74G)	pSS1354	this study
pNH605-P_{ADH}-OsTIR1(F74G,S210A)	pSS1649	this study
pNH604-P_{ADH}-OsTIR1(F74G)	pSS1361	this study
pNH605-P_{ADH}-OsTIR1	pSS1402	this study
pNH605-P_{ADH}-OsTIR1(F74A)	pSS1444	this study
pSH-EFIRES-B-AtAFB2-mCherry	pSS1063	Li et al., 2019
pRS405-P_{ADH}-AtAFB2	pSS1195	this study
pNH605-P_{ADH}-AtAFB2	pSS1446	this study
pNH605-P_{ADH}-AtAFB2(F74G)	pSS1447	this study
pNH605-P_{ADH}-AtAFB2(F74A)	pSS1448	this study
pRS303K-P_{GPD}-TagBFP	pSS348	Szoradi et al., 2018
pRS303K-P_{GPD}-sfGFP	pSS858	this study
pYTK021	pSS1494	Lee et al., 2015
pRS303K-P_{RNR1}-sfGFP	pSS1517	this study
pRS303K-P_{ADH}-sfGFP	pSS1516	this study
pRS303K-P_{GPD}-OsTIR1(F74G)	pSS1553	this study
pRS303K-P_{ADH}-OsTIR1(F74G)	pSS1554	this study
pRS303K-P_{RNR1}-OsTIR1(F74G)	pSS1555	this study
pRS303H-P_{ADH}-OsTIR1(F74G)	pSS1631	this study
pRS303N-P_{ADH}-OsTIR1(F74G)	pSS1632	this study
pNH605-P_{ADH}-GEM-P_{GAL}	pSS474	Schmidt et al., 2019
pRS303K-P_{GAL}-sfGFP	pSS1599	this study
pRS303K-P_{GAL}-sfGFP-P_{ADH}-GEM	pSS1601	this study
pRS303K-P_{GAL}-OsTIR1(F74G)-P_{ADH}-GEM	pSS1624	this study
pRS303H-P_{GPD}-TagBFP	pSS349	Szoradi et al., 2018
pRS305-HAC1-splicing reporter	pSS275	Pincus et al., 2010
pRS306K-P_{GPD}-Ubi-F-mCherry-sfGFP	pSS715	Khmelinskii et al., 2012

pRS306K-P_{GPD}-Ubi-R-mCherry-sfGFP	pSS725	Khmelinskii et al., 2012
pRS305-GEM-P_{GAL}-GFP-Pho8	pSS567	this study
pRS406-P_{TEF}-mCherry-Ubc6	pSS482	this study

Table S2. Yeast strains used in this study. GEM, GAL4-ER-Msn2; Neon, mNeonGreen; sfGFP, superfolder GFP; SR, splicing reporter.

Strain	Relevant genotype
SSY122	*ADE2 leu2-3,112 trp1-1 ura3-1 his3-11,15 MATa*
SSY3818	*Tef4-Neon::HIS3*
SSY3821	*Tef4-mAID-Neon::HIS3*
SSY4450	*Tef4-AID*-Neon::HIS3*
SSY3815	*Tef4-mIAA7-Neon::HIS3*
SSY4008	*Tef4-Neon::HIS3 leu2::*P_{ADH}*-OsTIR1-LEU2*
SSY4014	*Tef4-mAID-Neon::HIS3 leu2::*P_{ADH}*-OsTIR1-LEU2*
SSY4474	*Tef4-AID*-Neon::HIS3 leu2::*P_{ADH}*-OsTIR1-LEU2*
SSY4020	*Tef4-mIAA7-Neon::HIS3 leu2::*P_{ADH}*-OsTIR1-LEU2*
SSY4009	*Tef4-Neon::HIS3 leu2::*P_{ADH}*-OsTIR1(F74G)-LEU2*
SSY4015	*Tef4-mAID-Neon::HIS3 leu2::*P_{ADH}*-OsTIR1(F74G)-LEU2*
SSY4475	*Tef4-AID*-Neon::HIS3 leu2::*P_{ADH}*-OsTIR1(F74G)-LEU2*
SSY4021	*Tef4-mIAA7-Neon::HIS3 leu2::*P_{ADH}*-OsTIR1(F74G)-LEU2*
SSY4013	*Tef4-Neon::HIS3 leu2::*P_{ADH}*-AtAFB2(F74A)-LEU2*
SSY4019	*Tef4-mAID-Neon::HIS3 leu2::*P_{ADH}*-AtAFB2(F74A)-LEU2*
SSY4476	*Tef4-AID*-Neon::HIS3 leu2::*P_{ADH}*-AtAFB2(F74A)-LEU2*
SSY4025	*Tef4-mIAA7-Neon::HIS3 leu2::*P_{ADH}*-AtAFB2(F74A)-LEU2*
SSY4110	*Fba1-Neon::HIS3*
SSY4451	*Fba1-mAID-Neon::HIS3*
SSY4185	*Fba1-AID*-Neon::HIS3*
SSY4111	*Fba1-mIAA7-Neon::HIS3*
SSY4491	*Fba1-Neon::HIS3 leu2::*P_{ADH}*-OsTIR1-LEU2*
SSY4477	*Fba1-mAID-Neon::HIS3 leu2::*P_{ADH}*-OsTIR1-LEU2*
SSY4493	*Fba1-AID*-Neon::HIS3 leu2::*P_{ADH}*-OsTIR1-LEU2*
SSY4495	*Fba1-mIAA7-Neon::HIS3 leu2::*P_{ADH}*-OsTIR1-LEU2*
SSY4112	*Fba1-Neon::HIS3 leu2::*P_{ADH}*-OsTIR1(F74G)-LEU2*
SSY4478	*Fba1-mAID-Neon::HIS3 leu2::*P_{ADH}*-OsTIR1(F74G)-LEU2*
SSY4186	*Fba1-AID*-Neon::HIS3 leu2::*P_{ADH}*-OsTIR1(F74G)-LEU2*
SSY4113	*Fba1-mIAA7-Neon::HIS3 leu2::*P_{ADH}*-OsTIR1(F74G)-LEU2*
SSY4492	*Fba1-Neon::HIS3 leu2::*P_{ADH}*-AtAFB2(F74A)-LEU2*
SSY4479	*Fba1-mAID-Neon::HIS3 leu2::*P_{ADH}*-AtAFB2(F74A)-LEU2*
SSY4494	*Fba1-AID*-Neon::HIS3 leu2::*P_{ADH}*-AtAFB2(F74A)-LEU2*
SSY4496	*Fba1-mIAA7-Neon::HIS3 leu2::*P_{ADH}*-AtAFB2(F74A)-LEU2*
SSY4452	*Htb1-Neon::HIS3*
SSY4453	*Htb1-mAID-Neon::HIS3*
SSY4454	*Htb1-AID*-Neon::HIS3*
SSY4455	*Htb1-mIAA7-Neon::HIS3*
SSY4621	*Htb1-Neon::HIS3 leu2::*P_{ADH}*-OsTIR1-LEU2*
SSY4482	*Htb1-mAID-Neon::HIS3 leu2::*P_{ADH}*-OsTIR1-LEU2*
SSY4485	*Htb1-AID*-Neon::HIS3 leu2::*P_{ADH}*-OsTIR1-LEU2*
SSY4488	*Htb1-mIAA7-Neon::HIS3 leu2::*P_{ADH}*-OsTIR1-LEU2*
SSY4480	*Htb1-Neon::HIS3 leu2::*P_{ADH}*-OsTIR1(F74G)-LEU2*
SSY4483	*Htb1-mAID-Neon::HIS3 leu2::*P_{ADH}*-OsTIR1(F74G)-LEU2*
SSY4486	*Htb1-AID*-Neon::HIS3 leu2::*P_{ADH}*-OsTIR1(F74G)-LEU2*
SSY4489	*Htb1-mIAA7-Neon::HIS3 leu2::*P_{ADH}*-OsTIR1(F74G)-LEU2*

SSY4953	*Htb1-mIAA7-Neon::HIS3 leu2::*P_{ADH}*-OsTIR1(F74G,S210A)-LEU2*
SSY4481	*Htb1-Neon::HIS3 leu2::*P_{ADH}*-AtAFB2(F74A)-LEU2*
SSY4484	*Htb1-mAID-Neon::HIS3 leu2::*P_{ADH}*-AtAFB2(F74A)-LEU2*
SSY4487	*Htb1-AID*-Neon::HIS3 leu2::*P_{ADH}*-AtAFB2(F74A)-LEU2*
SSY4490	*Htb1-mIAA7-Neon::HIS3 leu2::*P_{ADH}*-AtAFB2(F74A)-LEU2*
SSY1049	*Sec63-Neon::HIS3*
SSY3608	*Sec63-mAID-Neon::HIS3*
SSY4141	*Sec63-AID*-Neon::HIS3*
SSY3812	*Sec63-mIAA7-Neon::HIS3*
SSY3830	*Sec63-Neon::HIS3 leu2::*P_{ADH}*-OsTIR1-LEU2*
SSY3833	*Sec63-mAID-Neon::HIS3 leu2::*P_{ADH}*-OsTIR1-LEU2*
SSY4497	*Sec63-AID*-Neon::HIS3 leu2::*P_{ADH}*-OsTIR1-LEU2*
SSY3835	*Sec63-mIAA7-Neon::HIS3 leu2::*P_{ADH}*-OsTIR1-LEU2*
SSY3831	*Sec63-Neon::HIS3 leu2::*P_{ADH}*-OsTIR1(F74G)-LEU2*
SSY3672	*Sec63-mAID-Neon::HIS3 leu2::*P_{ADH}*-OsTIR1(F74G)-LEU2*
SSY4142	*Sec63-AID*-Neon::HIS3 leu2::*P_{ADH}*-OsTIR1(F74G)-LEU2*
SSY3836	*Sec63-mIAA7-Neon::HIS3 leu2::*P_{ADH}*-OsTIR1(F74G)-LEU2*
SSY3832	*Sec63-Neon::HIS3 leu2::*P_{ADH}*-OsTIR1(F74A)-LEU2*
SSY3834	*Sec63-mAID-Neon::HIS3 leu2::*P_{ADH}*-OsTIR1(F74A)-LEU2*
SSY4498	*Sec63-AID*-Neon::HIS3 leu2::*P_{ADH}*-OsTIR1(F74A)-LEU2*
SSY3837	*Sec63-mIAA7-Neon::HIS3 leu2::*P_{ADH}*-OsTIR1(F74A)-LEU2*
SSY3852	*Sec63-Neon::HIS3 leu2::*P_{ADH}*-AtAFB2(F74A)-LEU2*
SSY3855	*Sec63-mAID-Neon::HIS3 leu2::*P_{ADH}*-AtAFB2(F74A)-LEU2*
SSY4499	*Sec63-AID*-Neon::HIS3 leu2::*P_{ADH}*-AtAFB2(F74A)-LEU2*
SSY3858	*Sec63-mIAA7-Neon::HIS3 leu2::*P_{ADH}*-AtAFB2(F74A)-LEU2*
SSY3864	*Vph1-Neon::HIS3*
SSY3865	*Vph1-mAID-Neon::HIS3*
SSY4139	*Vph1-AID*-Neon::HIS3*
SSY3813	*Vph1-mIAA7-Neon::HIS3*
SSY3866	*Vph1-Neon::HIS3 leu2::*P_{ADH}*-OsTIR1-LEU2*
SSY3869	*Vph1-mAID-Neon::HIS3 leu2::*P_{ADH}*-OsTIR1-LEU2*
SSY4508	*Vph1-AID*-Neon::HIS3 leu2::*P_{ADH}*-OsTIR1-LEU2*
SSY3872	*Vph1-mIAA7-Neon::HIS3 leu2::*P_{ADH}*-OsTIR1-LEU2*
SSY3867	*Vph1-Neon::HIS3 leu2::*P_{ADH}*-OsTIR1(F74G)-LEU2*
SSY3870	*Vph1-mAID-Neon::HIS3 leu2::*P_{ADH}*-OsTIR1(F74G)-LEU2*
SSY4140	*Vph1-AID*-Neon::HIS3 leu2::*P_{ADH}*-OsTIR1(F74G)-LEU2*
SSY3873	*Vph1-mIAA7-Neon::HIS3 leu2::*P_{ADH}*-OsTIR1(F74G)-LEU2*
SSY4956	*Vph1-mIAA7-Neon::HIS3 leu2::*P_{ADH}*-OsTIR1(F74G,S210A)-LEU2*
SSY3868	*Vph1-Neon::HIS3 leu2::*P_{ADH}*-OsTIR1(F74A)-LEU2*
SSY3871	*Vph1-mAID-Neon::HIS3 leu2::*P_{ADH}*-OsTIR1(F74A)-LEU2*
SSY4509	*Vph1-AID*-Neon::HIS3 leu2::*P_{ADH}*-OsTIR1(F74A)-LEU2*
SSY3874	*Vph1-mIAA7-Neon::HIS3 leu2::*P_{ADH}*-OsTIR1(F74A)-LEU2*
SSY3878	*Vph1-Neon::HIS3 leu2::*P_{ADH}*-AtAFB2(F74A)-LEU2*
SSY3881	*Vph1-mAID-Neon::HIS3 leu2::*P_{ADH}*-AtAFB2(F74A)-LEU2*
SSY4510	*Vph1-AID*-Neon::HIS3 leu2::*P_{ADH}*-AtAFB2(F74A)-LEU2*
SSY3884	*Vph1-mIAA7-Neon::HIS3 leu2::*P_{ADH}*-AtAFB2(F74A)-LEU2*
SSY3877	*Vph1-Neon::HIS3 leu2::*P_{ADH}*-AtAFB2(F74G)-LEU2*
SSY3880	*Vph1-mAID-Neon::HIS3 leu2::*P_{ADH}*-AtAFB2(F74G)-LEU2*
SSY4511	*Vph1-AID*-Neon::HIS3 leu2::*P_{ADH}*-AtAFB2(F74G)-LEU2*
SSY3883	*Vph1-mIAA7-Neon::HIS3 leu2::*P_{ADH}*-AtAFB2(F74G)-LEU2*

SSY3819	*Tom70-Neon::HIS3*
SSY3822	*Tom70-mAID-Neon::HIS3*
SSY4179	*Tom70-AID*-Neon::HIS3*
SSY3816	*Tom70-mIAA7-Neon::HIS3*
SSY3907	*Tom70-Neon::HIS3 leu2::P_{ADH}-OsTIR1-LEU2*
SSY3910	*Tom70-mAID-Neon::HIS3 leu2::P_{ADH}-OsTIR1-LEU2*
SSY4501	*Tom70-AID*-Neon::HIS3 leu2::P_{ADH}-OsTIR1-LEU2*
SSY3913	*Tom70-mIAA7-Neon::HIS3 leu2::P_{ADH}-OsTIR1-LEU2*
SSY3908	*Tom70-Neon::HIS3 leu2::P_{ADH}-OsTIR1(F74G)-LEU2*
SSY3911	*Tom70-mAID-Neon::HIS3 leu2::P_{ADH}-OsTIR1(F74G)-LEU2*
SSY4180	*Tom70-AID*-Neon::HIS3 leu2::P_{ADH}-OsTIR1(F74G)-LEU2*
SSY3914	*Tom70-mIAA7-Neon::HIS3 leu2::P_{ADH}-OsTIR1(F74G)-LEU2*
SSY3923	*Tom70-Neon::HIS3 leu2::P_{ADH}-AtAFB2(F74A)-LEU2*
SSY3926	*Tom70-mAID-Neon::HIS3 leu2::P_{ADH}-AtAFB2(F74A)-LEU2*
SSY4502	*Tom70-AID*-Neon::HIS3 leu2::P_{ADH}-AtAFB2(F74A)-LEU2*
SSY3915	*Tom70-mIAA7-Neon::HIS3 leu2::P_{ADH}-AtAFB2(F74A)-LEU2*
SSY4383	*his3::P_{RNR1}-sfGFP-kan*
SSY4382	*his3::P_{ADH}-sfGFP-kan*
SSY4385	*his3::P_{GPD}-sfGFP-kan*
SSY4712	*his3::P_{GAL}-sfGFP-GEM-kan*
SSY4440	*Sec63-mIAA7-Neon::HIS3 his3::P_{RNR1}-OsTIR1(F74G)-kan*
SSY4439	*Sec63-mIAA7-Neon::HIS3 his3::P_{ADH}-OsTIR1(F74G)-kan*
SSY4438	*Sec63-mIAA7-Neon::HIS3 his3::P_{GPD}-OsTIR1(F74G)-kan*
SSY4437	*Vph1-mIAA7-Neon::HIS3 his3::P_{RNR1}-OsTIR1(F74G)-kan*
SSY4436	*Vph1-mIAA7-Neon::HIS3 his3::P_{ADH}-OsTIR1(F74G)-kan*
SSY4435	*Vph1-mIAA7-Neon::HIS3 his3::P_{GPD}-OsTIR1(F74G)-kan*
SSY4434	*Fba1-mIAA7-Neon::HIS3 his3::P_{RNR1}-OsTIR1(F74G)-kan*
SSY4433	*Fba1-mIAA7-Neon::HIS3 his3::P_{ADH}-OsTIR1(F74G)-kan*
SSY4432	*Fba1-mIAA7-Neon::HIS3 his3::P_{GPD}-OsTIR1(F74G)-kan*
SSY4775	*Htb1-mIAA7-Neon::HIS3 his3::P_{RNR1}-OsTIR1(F74G)-kan*
SSY4774	*Htb1-mIAA7-Neon::HIS3 his3::P_{ADH}-OsTIR1(F74G)-kan*
SSY4773	*Htb1-mIAA7-Neon::HIS3 his3::P_{GPD}-OsTIR1(F74G)-kan*
SSY4793	*Tef4-mIAA7-Neon::HIS3 his3::P_{GAL}-OsTIR1(F74G)-GEM-kan*
SSY4794	*Vph1-mIAA7-Neon::HIS3 his3::P_{GAL}-OsTIR1(F74G)-GEM-kan*
SSY795	*his3::P_{GPD}-TagBFP-hph*
SSY3953	*his3::P_{GPD}-TagBFP-hph leu2::HAC1-SR-LEU2 trp1::P_{ADH}-OsTIR1(F74G)-TRP1*
SSY3954	*his3::P_{GPD}-TagBFP-hph leu2::HAC1-SR-LEU2 trp1::P_{ADH}-OsTIR1(F74G)-TRP1 Alg13-mIAA7-3xFLAG::kan*
SSY4275	*Sec63-Neon::HIS3 Rtn1-mCherry::nat leu2::P_{ADH}-OsTIR1(F74G)-LEU2 Alg13-mIAA7-3xFLAG::kan*
SSY4868	*Rpn1-mIAA7-ALFA::HIS3 leu2::P_{ADH}-OsTIR1(F74G)-LEU2*
SSY4884	*Rpn1-mIAA7-ALFA::HIS3 leu2::P_{ADH}-OsTIR1(F74G)-LEU2 ura3::Ub-R-tFT-kan*
SSY4886	*Rpn1-mIAA7-ALFA::HIS3 leu2::P_{ADH}-OsTIR1(F74G)-LEU2 ura3::Ub-F-tFT-kan*
SSY4894	*Rpn1-mIAA7-ALFA::HIS3 leu2::P_{ADH}-OsTIR1(F74G)-LEU2 ura3::Ub-R-tFT-kan pdr5Δ::nat*
SSY4896	*Rpn1-mIAA7-ALFA::HIS3 leu2::P_{ADH}-OsTIR1(F74G)-LEU2 ura3::Ub-F-tFT-kan pdr5Δ::nat*
SSY4879	*sec65-1 leu2::GEM-P_{GAL}-GFP-Pho8-LEU2 ura3::P_{TEF}-mCherry-Ubc6-URA3*
SSY4873	*Sec65-mIAA7-ALFA::HIS3 his3::P_{ADH}-OsTIR1(F74G)-kan leu2::GEM-P_{GAL}-GFP-Pho8-LEU2 ura3::P_{TEF}-mCherry-Ubc6-URA3*

Table S3. Plasmids and cognate forward and reverse primers for gene tagging. F2 primers consist of the 40-nucleotide sequence immediately upstream of the stop codon of a target gene (excluding the stop codon), followed by 5'-CGGATCCCCGGGTTAATTAA-3'. R1 primers consist of the reverse complement of the 40-nucleotide sequence immediately downstream of the stop codon of a target gene (excluding the stop codon), followed by 5'-GAATTCGAGCTCGTTTAAAC-3' (Longtine et al., 1998). S3 primers consist of the 42-nucleotide sequence immediately upstream of the stop codon of a target gene (excluding the stop codon), followed by 5'-CGTACGCTGCAGGTCGAC-3'. S2 primers consist of the reverse complement of the 41-nucleotide sequence immediately downstream of the stop codon of a target gene (excluding the stop codon), followed by 5'-ATCGATGAATTCGAGCTCG-3' (Janke et al., 2004).

**Plasmid**	**Alias**	**forward primer**	**reverse primer**
pFA6a-mAID-Neon-HIS3MX6	pSS1322	F2	R1 or S2
pFA6a-mAID-Neon-klTRP1	pSS1323	F2	R1 or S2
pFA6a-AID*-Neon-HIS3MX6	pSS1483	F2	R1 or S2
pFA6a-mIAA7-Neon-HIS3MX6	pSS1392	F2	R1 or S2
pFA6a-mIAA7-Neon-klTRP1	pSS1393	F2	R1 or S2
pFA6a-mIAA7-Neon-kanNP1	pSS1637	F2	R1 or S2
pFA6a-mIAA7-Neon-natNT2	pSS1639	S3	R1 or S2
pFA6a-mIAA7-Neon-hphNT1	pSS1638	S3	R1 or S2
pFA6a-mIAA7-Cherry-HIS3MX6	pSS1626	F2	R1 or S2
pFA6a-mIAA7-Cherry-klTRP1	pSS1625	F2	R1 or S2
pFA6a-mIAA7-Cherry-kanNP1	pSS1617	F2	R1 or S2
pFA6a-mIAA7-3xFLAG-HIS3MX6	pSS1475	F2	R1 or S2
pFA6a-mIAA7-3xFLAG-klTRP1	pSS1476	F2	R1 or S2
pFA6a-mIAA7-3xFLAG-kanNP1	pSS1616	F2	R1 or S2
pFA6a-mIAA7-ALFA-HIS3MX6	pSS1633	F2	R1 or S2
pFA6a-mIAA7-ALFA-klTRP1	pSS1634	F2	R1 or S2
pFA6a-mIAA7-ALFA-kanNP1	pSS1635	F2	R1 or S2

Table S4. Integrative expression plasmids for degron receptors. For genomic integration, expression plasmids need to be linearized by restriction digest.

Plasmid	Alias	Restriction digest with
pRS405-P_{ADH}-OsTIR1(F74G)	pSS1330	AgeI or EcoRI
pRS406-P_{ADH}-OsTIR1(F74G)	pSS1331	StuI or BstBI
pNH605-P_{ADH}-OsTIR1(F74G)	pSS1354	PmeI
pNH605-P_{ADH}-OsTIR1(F74G,S210A)	pSS1649	PmeI
pNH604-P_{ADH}-OsTIR1(F74G)	pSS1361	PmeI
pNH605-P_{ADH}-OsTIR1	pSS1402	PmeI
pNH605-P_{ADH}-OsTIR1(F74A)	pSS1444	PmeI
pRS405-P_{ADH}-AtAFB2	pSS1195	AflII or AjuI
pNH605-P_{ADH}-AtAFB2	pSS1446	PmeI
pNH605-P_{ADH}-AtAFB2(F74G)	pSS1447	PmeI
pNH605-P_{ADH}-AtAFB2(F74A)	pSS1448	PmeI
pRS303K-P_{GPD}-OsTIR1(F74G)	pSS1553	NheI/AfeI (double digest)
pRS303K-P_{ADH}-OsTIR1(F74G)	pSS1554	NheI/AfeI (double digest)
pRS303K-P_{RNR1}-OsTIR1(F74G)	pSS1555	NheI/AfeI (double digest)
pRS303H-P_{ADH}-OsTIR1(F74G)	pSS1631	NheI/AfeI (double digest)
pRS303N-P_{ADH}-OsTIR1(F74G)	pSS1632	NheI/AfeI (double digest)
pRS303K-P_{GAL}-OsTIR1(F74G)-GEM	pSS1624	NheI/AfeI (double digest)
